# Global Impact of Aging on the Hemodynamic Response Function in the Gray Matter of Human Cerebral Cortex

**DOI:** 10.1002/hbm.70100

**Published:** 2024-12-18

**Authors:** Nooshin J. Fesharaki, Amanda Taylor, Keisjon Mosby, Ruosha Li, Jung Hwan Kim, David Ress

**Affiliations:** ^1^ Department of Neurosurgery University of Texas Health Science Center Houston Texas USA; ^2^ Department of Neuroscience, High Resolution Brain Imaging Lab Baylor College of Medicine Houston Texas USA

**Keywords:** BOLD fMRI, healthy aging, hemodynamic response function, whole‐brain analysis

## Abstract

In functional magnetic resonance imaging, the hemodynamic response function (HRF) is a stereotypical response to local changes in cerebral hemodynamics and oxygen metabolism due to briefly (< 4 s) evoked neural activity. Accordingly, the HRF is often used as an impulse response with the assumption of linearity in data analysis. In cognitive aging studies, it has been very common to interpret differences in brain activation as age‐related changes in neural activity. Contrary to this assumption, however, evidence has accrued that normal aging may also significantly affect the vasculature, thereby affecting cerebral hemodynamics and metabolism, confounding interpretation of fMRI cognitive aging studies. In this study, use was made of a multisensory task to evoke the HRF in ~87% of cerebral cortex in cognitively intact adults with ages ranging from 22 to 75 years. This widespread activation enabled us to investigate age trends in the spatial distributions of HRF characteristics within the majority of cortical gray matter, which we termed as global age trends. The task evoked both positive and negative HRFs, which were characterized using model‐free parameters in native‐space coordinates. We found significant global age trends in the distributions of HRF parameters in terms of both amplitudes (e.g., peak amplitude and contrast‐to‐noise ratio) and temporal dynamics (e.g., full‐width‐at‐half‐maximum). Our findings offer insight into how age‐dependent changes affect neurovascular coupling and show promise for use of HRF parameters as non‐invasive indicators for age‐related pathology.

## Graphical abstract

(A) The SAST (speeded audiovisual sequence‐following task) evokes an HRF (hemodynamic response function). (B) HRF parameters such as full‐width‐at‐half‐maximum (FWHM) are calculated. (C) Example HRF parameter maps on native‐space surfaces. (D) Corresponding HRF parameter distributions. (E) Parameter spatial means and standard deviations were correlated with age; *R* is correlation coefficient.

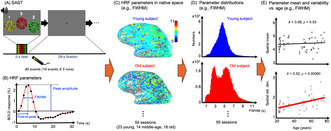

## 1 Introduction

Functional magnetic resonance imaging (fMRI) signals based on blood oxygenation level dependent (BOLD) contrast arise from local changes in cerebral hemodynamics in response to neural activity, through a mechanism known as neurovascular coupling (NVC) (Ogawa et al. 1992). A stereotypical BOLD response evoked by brief (< 4 s) stimuli is known as hemodynamic response function (HRF) (Boynton et al. 1996; Taylor, Kim, and Ress 2018, 2022). Models of the HRF predict that it is formed by local changes in cerebral blood flow (CBF) and oxygen metabolism (CMRO_2_) (Buxton 2012; Kim and Ress 2016), and therefore, has potential to indicate neurovascular and neurometabolic integrity in the human brain (Bonakdarpour, Parrish, and Thompson 2007; Biessmann et al. 2012).

In previous research, detailed measurements of the microvascular architecture in rodents revealed a fairly regular hexagonal mesh of pial arterioles overlaying the gray matter (GM) in the arachnoid space (Blinder et al. 2013). Based on the features of this mesh, we developed our earlier linear network modeling of the HRF (Kim and Ress 2016; Ress et al. 2009; Kim et al. 2013). Indeed, our previous model identified two types of energy during NVC: (1) kinetic energy in the form of moving blood and (2) energy stored due to vascular compliance. The cerebral microvasculature has been measured non‐invasively in the human brain using diffuse correlation spectroscopy, showing a substantial compliance: *C*_*p*_ = 4.8 × 10^−4^ mL/mmHg/min/100 g (Baker et al. 2017). This compliance enables the pial mesh to store energy in the elasticity of the pial arterioles. When task‐ or stimulus‐evoked neural activity occurs, a wave of vasodilation (*proximal integration*) (Itoh and Suzuki 2012) propagates from the capillary parenchyma to this pial mesh, creating a pressure fluctuation that drives the HRF. In our HRF modeling, we indicated that while competition between CBF and CMRO_2_ responses to brief stimulation can give rise to the BOLD HRF, CBF is usually the dominant component corresponding to NVC. This CBF response is mediated by the described microvascular architecture and is well‐modeled by an underdamped oscillatory sinusoidal kernel.

Evidence from animal models has accumulated showing that aging is associated with multifaceted vascular changes at molecular, cellular, and structural levels (Tarantini et al. 2018; Yabluchanskiy et al. 2021; Zimmerman et al. 2021). For instance, structural integrity of cerebral vessels became compromised in aged rodent models (Yabluchanskiy et al. 2021; Li et al. 2018; Kang et al. 2016). Aging may potentially result in dysfunction of endothelial cells as well as proliferation and migration of vascular smooth muscle cells in arterioles (Donato, Machin, and Lesniewski 2018). Increased vascular wall thickness but decreased vascular elasticity, would ultimately affect conductance, causing vasomotility dysfunction (Yabluchanskiy et al. 2021; Zimmerman et al. 2021). Recently, impairment of pericyte dynamics has been attributed into capillary remodeling in aged mice (Berthiaume et al. 2022; Ding et al. 2020). Aging has also been linked to vascular rarefaction and increased arterial tortuosity (Tarantini et al. 2016; Springo et al. 2015), and may increase large‐artery stiffness, amplifying the dissipation of pulsatile energy in the heavily vascularized brain, causing microvasculature damage (Harvey, Montezano, and Touyz 2015; Wang et al. 2018; Jennings et al. 2021).

Recent research in humans has also suggested that aging may affect NVC (Abdelkarim et al. 2019; Turner et al. 2022). Imaging techniques such as arterial spin labeling MRI and positron emission tomography have quantified CBF and CMRO_2_ to potentially enable calculation of the NVC ratio in older versus younger adults (Abdelkarim et al. 2019; Turner et al. 2022; Aanerud et al. 2012; De Vis et al. 2015; Camargo and Wang 2021; Catchlove et al. 2018). A large body of evidence supports that aging correlates with reductions in both regional (e.g., temporal and parietal lobes) CBF and CMRO_2_, frequently with greater reductions in CBF than in CMRO_2_ (Aanerud et al. 2012; De Vis et al. 2015; Camargo and Wang 2021; Alisch et al. 2021; Asllani et al. 2009; Ibaraki et al. 2010; Lu et al. 2011; Krishnamurthy et al. 2020). However, both CBF and CMRO_2_ may remain intact in aged GM if the effect of brain atrophy is controlled (Catchlove et al. 2018). The possibility for regional increases in both CBF and CMRO_2_ with age has also been shown (Lee et al. 2009; Peng et al. 2014). Altogether, age‐related changes in human resting CBF and CMRO_2_ remain controversial.

Using fMRI, BOLD signal variations in elderly subjects are typically associated with age‐related changes in neural activity based on the assumption that NVC is age‐invariant, and thus, commonly using a canonical HRF for BOLD‐signal analysis (Aizenstein et al. 2004; La et al. 2016; Berghuis et al. 2019; Mayhew et al. 2022). However, this assumption and use of canonical HRF are likely invalid with advancing age (West et al. 2019). Clearly, there is a need to evaluate how NVC and the HRF change with aging. For example, the recent report by Krishnamurthy, et al. that age‐induced changes in language‐task BOLD HRF could be explained with corresponding changes in regional neurochemicals (Krishnamurthy et al. 2022).

There have been several age‐related studies of the HRF. Recently, West et al. (2019) regionally explored the characteristics of the BOLD HRF estimated using flexible basis sets to avoid assumptions on the shape of the HRF (West et al. 2019). They found significant differences in HRF parameters, particularly an increased time‐to‐peak (TTP) and an decreased peak amplitude for older versus younger subjects. Their analysis, however, was restricted to a set of fairly coarse, atlas‐defined brain regions. Contrarily, some studies have reported earlier TTP with age (Huettel, Singerman, and McCarthy 2001). Still others have also shown later TTP in older individuals (Taoka et al. 1998; Handwerker et al. 2007). Another study reported no significant timing changes with age (Brodtmann et al. 2003). Other age‐related HRF studies have concentrated on amplitude differences. Age‐related decreases in amplitude in response to visual stimuli have been frequently reported (Handwerker et al. 2007; Ross et al. 1997; Tekes et al. 2005; Ances et al. 2009) and similarly for motor tasks (Tekes et al. 2005; Buckner et al. 2000; Hesselmann et al. 2001). However, others have observed no significant age differences in amplitude (Aizenstein et al. 2004; Huettel, Singerman, and McCarthy 2001; Brodtmann et al. 2003; D'Esposito et al. 1999; Ward, Swayne, and Newton 2008).

Inconsistency in studies of age‐related changes in the HRF could results from two main limitations. First, limited regional activation, so only a small subset of brain response was evaluated. Second, use of coarse spatial resolution that cannot resolve the GM, confounding interpretation because of partial volume effects. Notably, HRF dynamics and amplitude vary between GM, white matter, and superficial vascular regions, with the highest reliability in GM (Kim and Ress 2017).

Notably, the need remains to fully characterize age‐related changes in HRF dynamics and amplitudes across the whole of cerebral cortex GM. This study addresses this need by investigating age‐dependent changes in HRF characteristics “globally,” that is, in the spatial distributions of HRF parameters across majority of the cortex. Such a study is important for three reasons. First, this knowledge is needed to interpret age‐related neural changes to various tasks and stimuli. Second, such work can help us better understand how age affects neurovascular and neurometabolic coupling. Most importantly, methods that broadly evoke the HRF across the brain have potential to provide a means to detect age‐related changes in brain function and pathology. In particular, predictions from our previously developed linear‐network modeling of the pial mesh suggests that age‐related disruptions, such as arterial rarefaction and hyalinosis, could cause changes in HRF dynamics, resulting in both faster and slower dynamics. We report experiments that evaluate this hypothesis.

Here, we evoked the HRF across the majority of cerebral cortex using the Speeded Audiovisual Sequence‐following Task (SAST). The evoked HRFs have stereotypical amplitudes and stable dynamics in young healthy brains (Taylor, Kim, and Ress 2018), and are stable over time frames of up to 3 months (Taylor, Kim, and Ress 2022). We acquired the HRF data using a simple, time‐locked averaging approach, which provides strong contrast‐to‐noise ratio (CNR) across the majority of cortex by rejecting temporally structured physiological nuisance and also avoids the need to assume linearity to measure the HRFs. Our previous modeling work (Kim and Ress 2016; Kim et al. 2013), and subsequent measurements (Kim et al. 2019), established that the dynamics of HRF are derived mainly from CBF, with only weakly modulatory effects from CMRO_2_. Thus, these broadly evoked HRFs in healthy young brains are largely diagnostic of NVC, and provide a basis for comparison, a “fingerprint” against which age‐related changes can be compared. As a first step in developing such methods, we used the SAST to evoke HRFs across cortex in 55 sessions that include subjects aged 22–75 years. We then investigated how the distributions of HRF amplitudes and dynamics across cortex change as a function of age. We found significant global effects in both amplitudes and dynamics. Using predictions from our previously developed modeling, we then aimed to conceptually connect the changes in HRF with known age‐related microvascular disruptions.

## 2 Methods

### 2.1 Participants

A cohort of 37 healthy subjects (ages: 22–75 years) with no history of neurological diseases was recruited for this study. Older subjects (age > 65) performed a Mini Mental State Evaluation and were only included if their score was ≥ 24. Of those, 23 subjects performed two scanning sessions in the same day separated by 3 h; this data were part of a study of HRF temporal stability published previously (Taylor, Kim, and Ress 2022). For the other subjects, only one session was collected, thereby yielding a total number of 60 datasets. Five sessions were then excluded from analysis due to excessive motion or low CNR. From the remaining 34 subjects (20 female), we analyzed 55 scanning sessions including 23 young (22–39 years, mean: 26.9 years, 8 male), 14 middle‐age (40–59 years, mean: 52.4 years, 12 male), and 18 older (60–75 years, mean: 65.9 years, 5 male) adults. These age groups were chosen as a convenient 3‐way split that roughly balances the available pool of subjects and sessions. For each subject, informed consent form was obtained at the time of first enrollment in the study. All experimental procedures were performed according to a protocol reviewed and approved by the Baylor College of Medicine Institutional Review Board, with review based on the principles of the Belmont Report. Prior to data collection, each subject was trained on the task and practiced the task using a mock scanner while their performance was validated. All subjects were also screened for MRI safety.

### 2.2 Experimental Tasks

**FIGURE 1:**
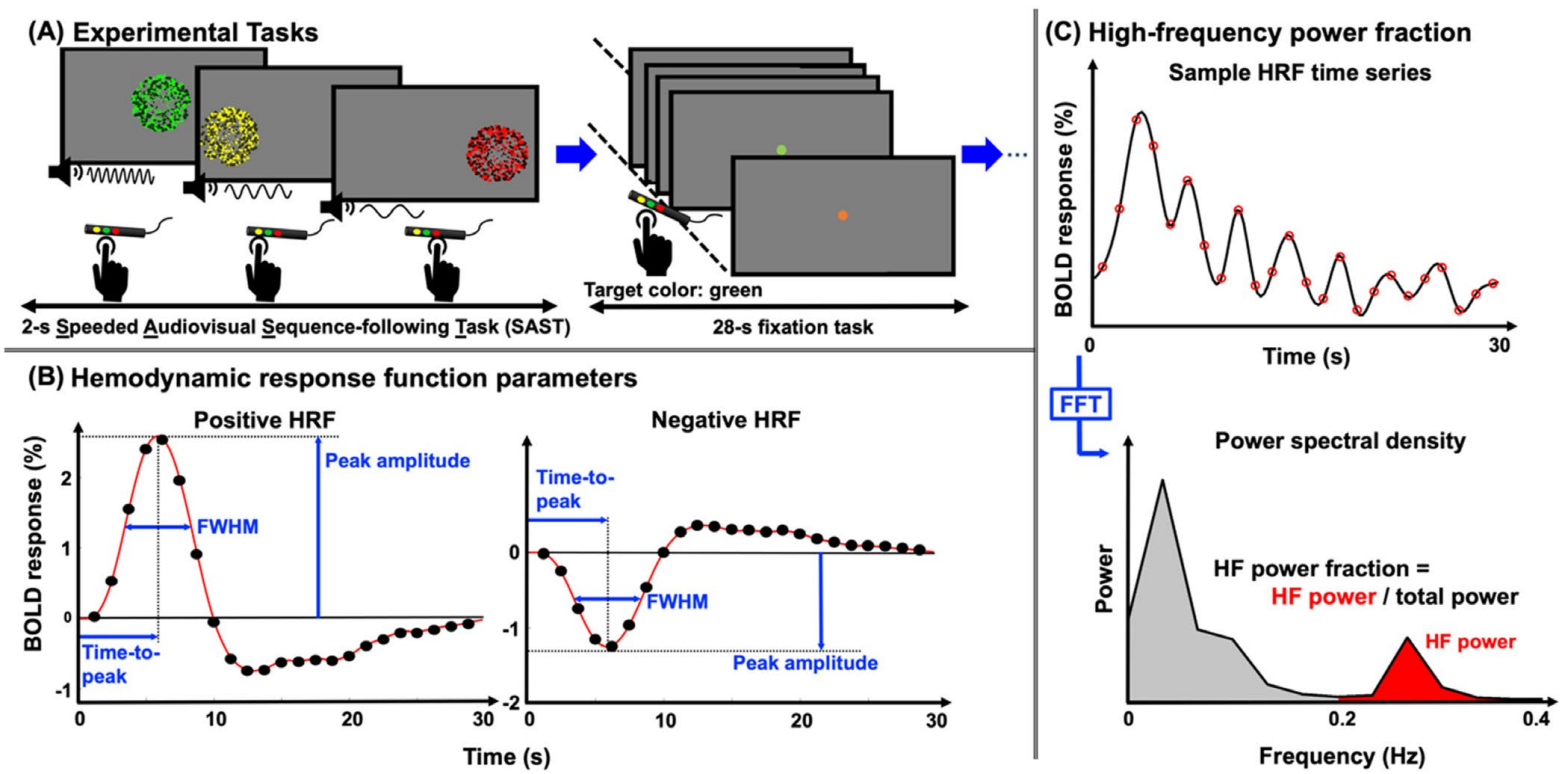
(A) Schematic shows the experimental task. A single HRF event consists of the 2‐s SAST and the 28‐s resting period fixation task. (B) HRF parameters are extracted from positive and negative BOLD responses including HRF peak amplitude, peak time, and full‐width‐at‐half‐maximum (FWHM). (C) High‐frequency (HF) power fraction is calculated as the ratio of high‐frequency (> 0.2 Hz) power to total power.

For each subject, HRFs were evoked from an event‐related‐design experiment, including epochs of a 2‐s SAST followed by a 28‐s non‐demanding fixation task (Figure 1A). The tasks were simple, and all subjects were readily trained. During each scan, subjects were asked to relax and stay as still as possible. They were cued by a change of fixation dot color to white‐on‐black color for 0.5 s, signaling them to expect the presentation of an audio‐visual stimulus for the next 2 s. The audio‐visual stimulus was a consecutive sequence of three “fireworks.”

Each “fireworks” was a patch of flickering (6 Hz) colored dots in either yellow, green, or red, accompanied by a specific sound; Each flickering‐colored‐dot patch was presented for 667 ms in one of three circular (5° radius) regions uniformly and horizontally distributed across the width of the screen. Vertical positions were randomly varied over a ± 3° range. The spatial order of presentation of each colored‐dot patch was random, without sequential repetition. Although presentation order varied, the patch colors and associated positions were fixed: yellow was always on the left, green in the center, and red on the right. Each of these colored‐dot patches was accompanied by an audio stimulus of bandpass‐filtered white noise: medium pitch during yellow dots, low pitch during red dots, and high pitch during green dots. Subjects were instructed to follow each firework with eye movements and quickly press buttons in the order corresponding to the color of the fireworks. To accommodate the slower reaction times of some (6) of the older subjects, the audio‐visual stimulus consisted of a sequence of only two fireworks during the same 2‐s interval, so the subjects needed to make two button presses in correspondence with the color of the randomly‐presented fireworks.

After the 2‐s stimulus presentation, subjects were asked to keep their gaze fixated on a central fixation dot (0.15° diameter) for 28 s and perform a non‐demanding fixation task to retain a low level of attention between audio‐visual stimuli. During the fixation period, subjects were asked to attend the fixation dot (0.15° diameter), which changed color every 0.6 s. Subjects were instructed to press a button upon the appearance of a single target color (yellow), which appeared on average every 6 s (Taylor, Kim, and Ress 2018). During each 2‐s presentation of three fireworks, reaction time, and response accuracy were recorded for each of the 2 or 3 times the subject pressed a button corresponding to the color of the firework.

Each 2‐s audiovisual stimulus sequence and following 28‐s fixation period constitute a 30‐s HRF measurement. For each subject, 16 HRFs were collected in each of 5 runs, yielding a total of 80 HRF measurements per session.

### 2.3 Magnetic Resonance Imaging Protocol

Imaging was performed on a 3‐T MAGNETOM Trio MRI scanner (Siemens Healthcare, Erlangen, Germany) using a 32‐channel RF head coil. FMRI data use an SMS‐accelerated echo‐planar imaging (EPI) sequence (Breuer et al. 2005; Setsompop et al. 2012) with TR = 1.25 s, TE = 30 ms, GRAPPA = 2, SMS = 3, 2‐mm square pixels on 60 2‐mm slices. During functional runs, stimulus timing aligns with the TR of the scanner (24 TRs per HRF).

During each session, a T1‐weighted volume aligned with the functional slice prescription was acquired before and after collecting functional images using a 3D FLASH sequence with minimum TE and TR, 15° flip angle, 256‐mm FOV, 64 slices, 2‐mm slice thickness, and 1‐mm inplane pixel size. There images were used to align the functional data to a high‐resolution T1‐weighted reference volume. The reference volume was obtained for each subject using a high‐resolution (0.8‐mm isotropic voxels) MP‐RAGE sequence with TR = 2300 ms, T1 = 900 ms, flip angle = 9°, 2 repetitions. The anatomy was then segmented into gray and white matter using FreeSurfer (Dale, Fischl, and Sereno 1999) with procedures to maintain native spatial resolution (Collins et al. 1994; Fischl, Sereno, and Dale 1999). Depth coordinates, normalized to local gray‐matter thickness, were also computed from the FreeSurfer segmentation and then used in a morphing procedure to create a normalized cortical depth map for each subject's reference volume (Kim and Ress 2017). This depth map normalized the GM thickness to a range of 0–1, with 0 assigned to the gray/white‐matter interface and 1 to the pial surface. The normalized depth map also provided streamlines that uniquely associated all GM voxels with the white‐matter surface, which were used later for the depth‐averaging analysis.

### 2.4 Data Analysis

For each fMRI scan, images were compensated for slice acquisition timing and for the effects of head motion using a robust expectation–maximization method (Nestares and Heeger 2000). Next, the motion‐corrected functional data were then brought into spatial alignment with each other and finally registered to a high‐resolution reference volume anatomy using the same intensity‐based alignment algorithm applied to the T1‐weighted inplane images collected in each session. Low‐frequency temporal baseline drifts were also removed from the time series, and the data were corrected for spatial variations due to receiver‐coil‐array inhomogeneity (Ress et al. 2007).

For each subject, the time series data were depth‐averaged across the GM thickness using their normalized depth map. To minimize partial volume effects, this was done by averaging the functional data of voxels located within the normalized depth range of 0.2–0.8. The averaged time series data, each containing the 80 HRF events, were then mapped onto the vertices of the white‐matter surface to aid visualization (Kim and Ress 2017).

The surface data were also smoothed with an 8‐mm‐full‐width‐at‐half‐maximum (FWHM) Gaussian kernel using surface‐manifold coordinates. Larger clusters (> 36 mm^2^) of negative HRFs (nHRFs) were smoothed separately to avoid mixing between nHRFs and positive HRFs (pHRFs). Both full‐resolution and smoothed time series data were analyzed for age‐related effects by averaging across HRF events and parameterizing them as described in detail below.

For each subject, the depth‐averaged data were censored for excessive motion, so that any of the 80 HRF events (16 events per scan × 5 scans) with head motion exceeding 2 mm/TR were removed from further analysis. The events that survived head motion were then averaged together to create an HRF at each surface vertex. Baseline correction was performed for each HRF by subtracting a zero baseline, estimated as the average of its first and last time points. We then used parameterization to characterize each HRF with a set of amplitude and timing parameters. Amplitude parameters, peak amplitude CNR, and high‐frequency power fraction (HFPF) were calculated first. Next, for temporal parameterization, each HRF's time series was upsampled to an interval of 0.1 s using spline interpolation, resulting in 300 time points. Two more parameters were then extracted from each HRF (Figure 1B,C): TTP, and FWHM. Peak amplitude was defined as the largest value within the first 2–14 s time window. This time interval was chosen to control for outliers caused by occasional noise in the time series. The HRF's CNR was calculated as the ratio of its peak amplitude (before upsampling) to its standard error of the mean (SEM) across the events. HFPF was also calculated only on the time series before upsampling. It was defined as the ratio of the power in the upper half of the frequency range, 0.2–0.4 Hz (Nyquist frequency is 0.4 Hz) to the total power (Figure 1C). Finally, to characterize the global tuning of HRFs across cortex for each subject, we evaluated the dependence of peak amplitude or HFPF on FWHM. These tuning curves were obtained by summing peak amplitudes or HFPF within small ranges of FWHM across the domain of 1–11 s, thus providing two further measures of hemodynamic energy as a function of HRF time‐scale. These tuning curves were then normalized by their peak value to permit comparisons among subjects. The FWHM width of the tuning curves, a tuning width, was used as a single parameter for evaluation as a function of age.

Based on the sign of the peak amplitudes, the HRFs were divided into pHRFs and nHRFs. For both, the spatial mean and standard deviation of all parameters, as well as CNR and HFPF were computed. We chose only strong HRFs with CNR > 3 for analysis. We then performed regression analysis to assess statistically significant (*p* < 0.05) correlations between HRF parameters and age. Because subjects had either one or two sessions, regressions were performed using a mixed‐effects model taking subject ID as a random effect.

## 3 Results

**FIGURE 2:**
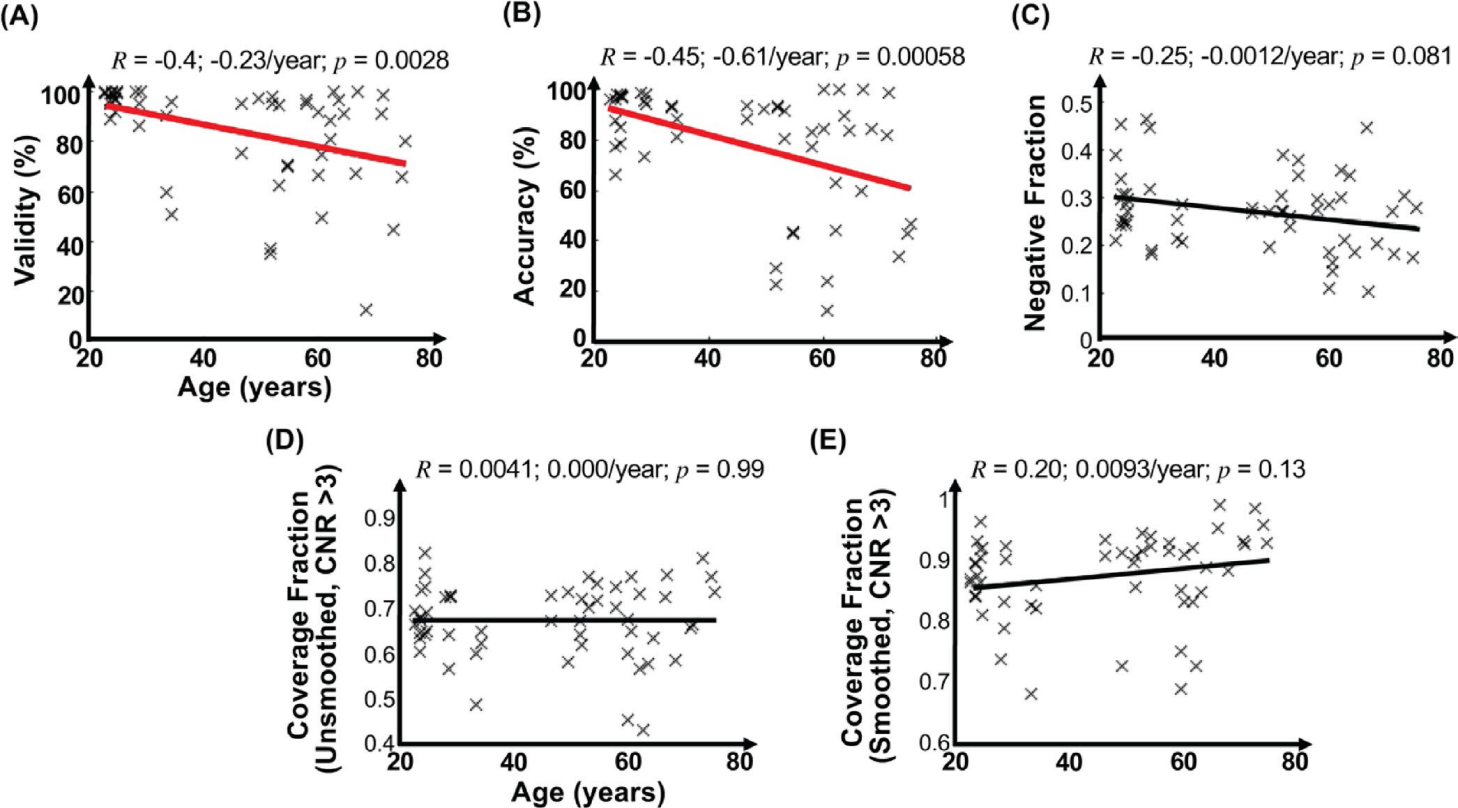
Correlations between age and (A) task‐performance validity, (B) task‐performance accuracy, (C) the fraction of cortex with negative HRFs, (D) the fraction of cortical coverage (contrast‐to‐noise ratio (CNR) > 3), and (E) the fraction of cortical coverage (CNR > 3) with 8‐mm full‐width‐at‐half‐maximum smoothing (CNR > 3). Pearson correlation coefficients (*R*) are shown for each feature. Significant (*p* < 0.05) correlations are marked by thicker regression lines.

Task performance was characterized by validity and accuracy. Valid tasks were those in which the subject pressed one of the assigned three buttons during the 2‐s stimulus period. Average validity was 93% ± 10%. Accuracy, assessed for valid events, was the fraction of events where the subject pressed the correct button. Average accuracy was 79% ± 24%. Validity and accuracy decrease significantly with age (*R* = −0.40, and *R* = −0.45, respectively, Figure 2A,B). Older subjects tended to respond too slowly, reducing both validity and accuracy. For the older subjects, average validity and accuracy were 88.3% and 67.5%, respectively. For correct trials, reaction time also significantly increased with age (Figure S1).

At full resolution, the SAST effectively evoked strong HRFs (CNR > 3) across the majority of cerebral cortex. Coverage, the fraction of cortex with CNR > 3, range was 42%–83%, mean of 68% ± 8%, where the variability noted is the SEM. Coverage was not affected by age (Figure 2D). However, smoothing did increase cortical coverage substantially (Figure 2E), with a coverage range of 69%–97%, mean of 87% ± 7%. Consistent with our previous work (Taylor, Kim, and Ress 2018), a subset of activated cortex responded with nHRFs (negative fraction), with a range of 12%–47%, mean 27% ± 8% across sessions. Additionally, negative fraction decreased with age, but the trend was not significant (*R* = −0.25, *p* = 0.081) (Figure 2C).

**FIGURE 3:**
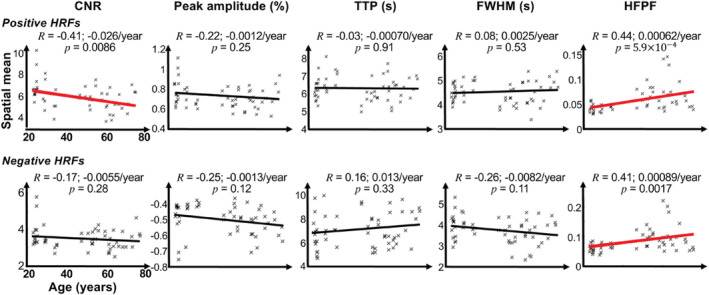
Correlations between age and the spatial mean of contrast‐to‐noise ratio (CNR), peak amplitude, time‐to‐peak (TTP), full‐width‐at‐half‐maximum (FWHM), and high‐frequency power fraction (HFPF) for top, positive HRFs (pHRFs), and bottom, negative HRFs (nHRFs). Pearson correlation coefficients (*R*) are shown for each HRF parameter. Significant (*p* < 0.05) correlations are marked by thicker, red regression lines.

**FIGURE 4:**
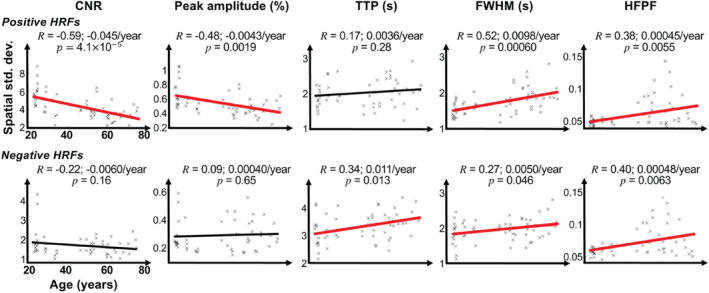
Correlations between age and the spatial standard deviation of contrast‐to‐noise ratio (CNR), peak amplitude, time‐to‐peak (TTP), full‐width‐at‐half‐maximum (FWHM), and high‐frequency power fraction (HFPF) for top, positive HRFs (pHRFs), and bottom, negative HRFs (nHRFs). Pearson correlation coefficients (*R*) are shown for each HRF parameter. Significant (*p* < 0.05) correlations are shown by thicker, red regression lines.

**FIGURE 5:**
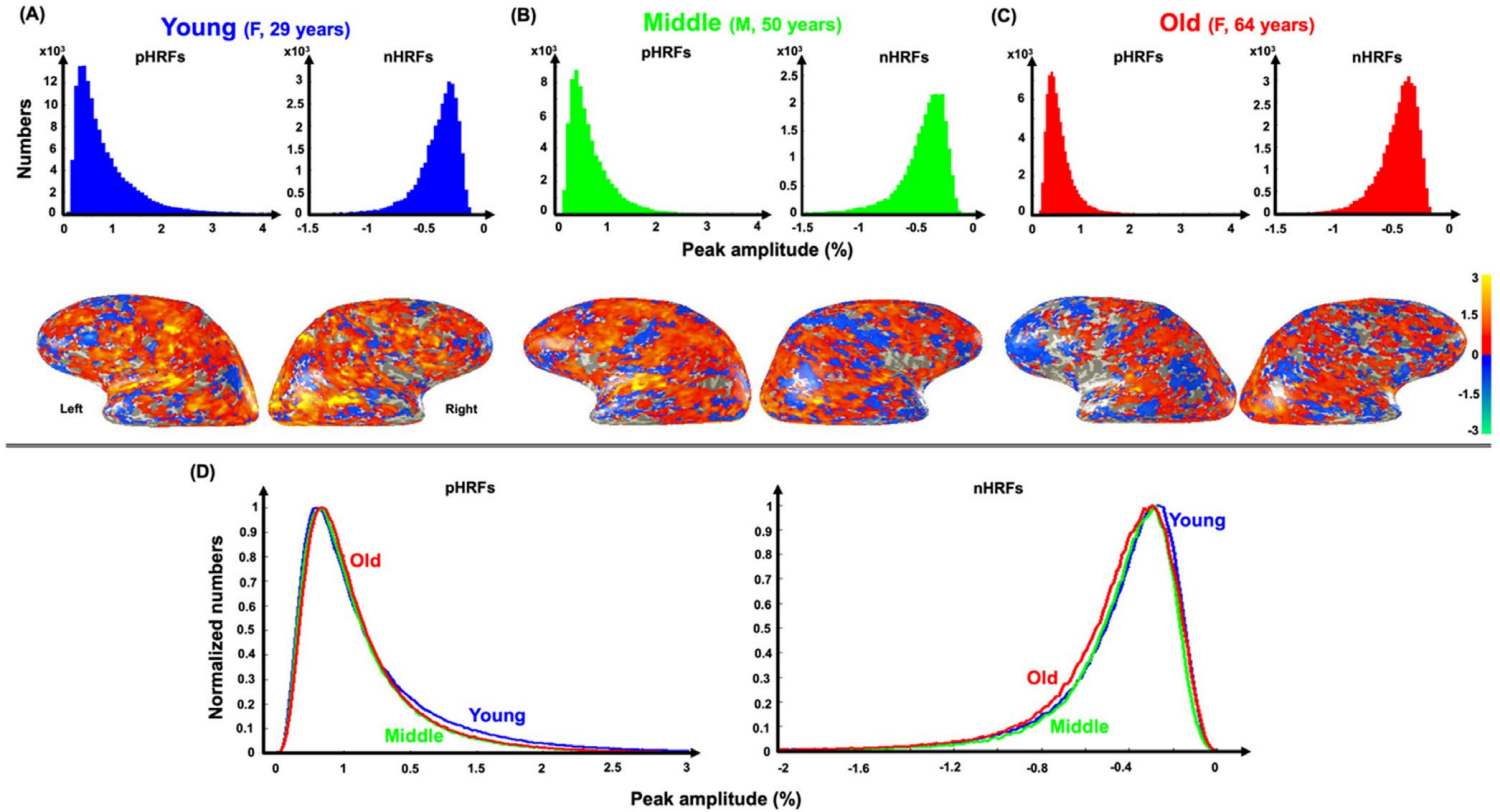
Example peak amplitude distributions of positive strong (CNR > 3) HRFs (pHRFs) and negative, strong (CNR > 3) HRFs (nHRFs) of (A) 29‐year‐old female, (B) 50‐year‐old male, and (C) 64‐year‐old female. (D) Mean normalized distributions of peak amplitude for pHRFs (left) and nHRFs (right) are shown for young (23 subjects, blue), middle‐age (14 subjects, green), and older (18 subjects, red) groups. CNR: Contrast‐to‐noise ratio.

**FIGURE 6:**
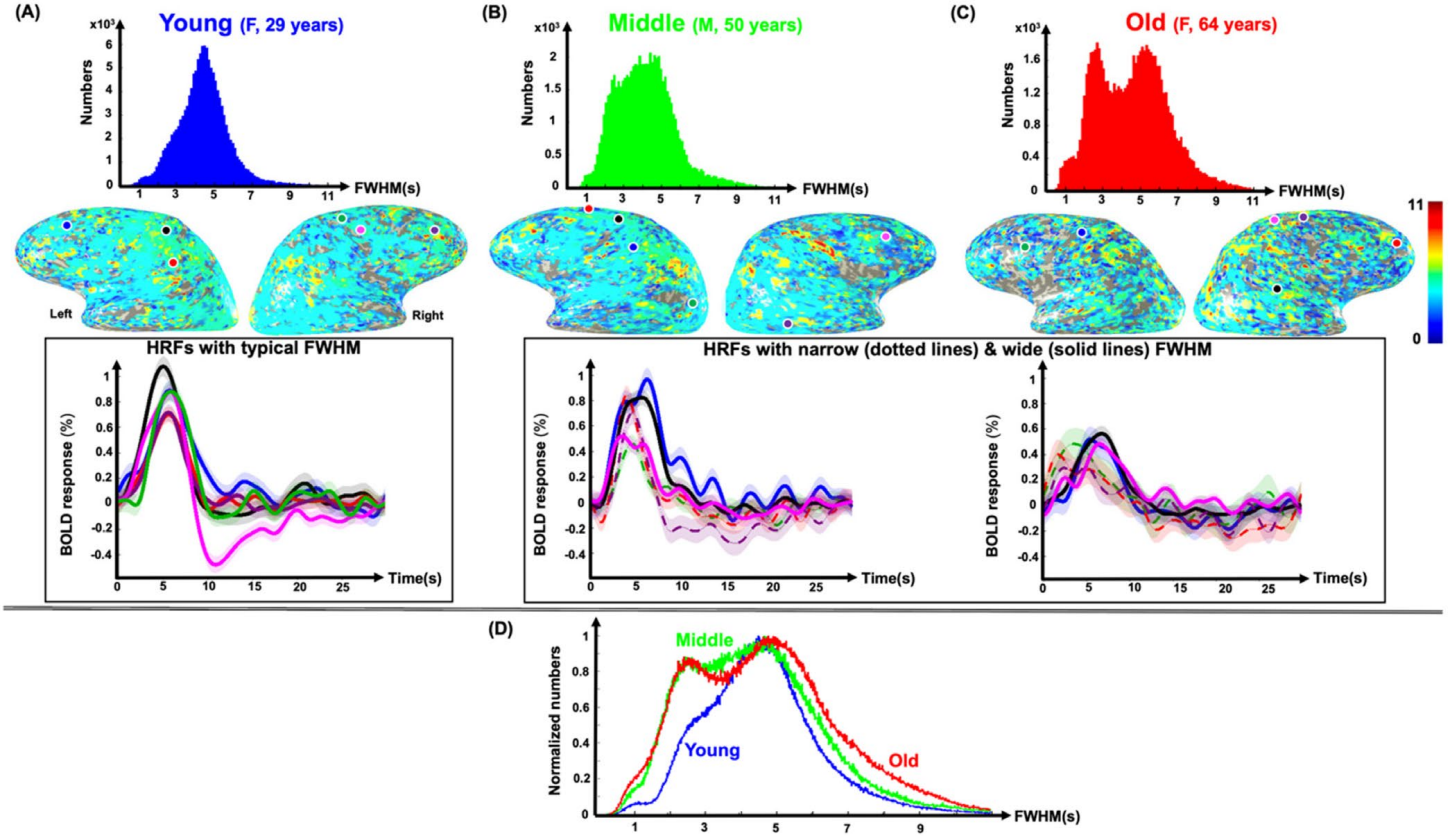
Example full‐width‐at‐half‐maximum (FWHM) distributions of positive, strong (contrast‐to‐noise ratio (CNR) > 3) HRFs (each averaging over ~80 events) of (A) 29‐year‐old female, (B) 50‐year‐old male, and (C) 64‐year‐old female. Corresponding HRF FWHM maps overlaid on gray‐white interface surfaces are shown below each distribution. Average HRFs (5‐mm gray‐matter disk) in six example ROIs for each subject are shown in the next row. For middle‐aged and older subjects, HRFs with example wide and narrow FWHM are shown by solid and dotted lines, respectively. Shaded regions show SEM (standard error of mean). (D) Mean normalized distributions of FWHM for strong, positive HRFs are shown for young (23 sessions, blue), middle‐age (14 sessions, green), and older (18 sessions, red) groups.

**FIGURE 7:**
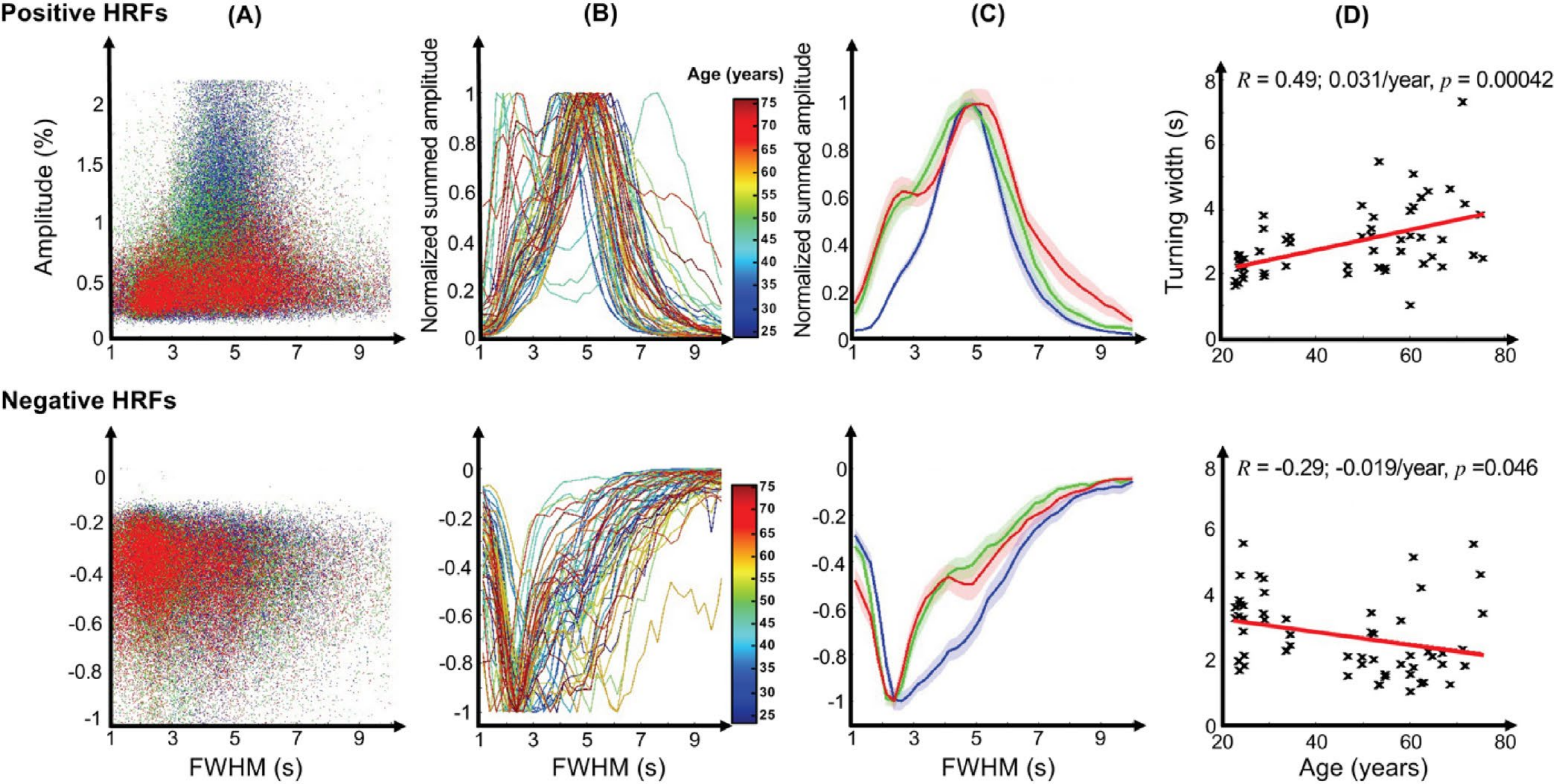
HRF tuning curves; upper row shows results for positive HRFs (pHRFs), lower row for negative HRFs (nHRFs). (A) Scatter plots of peak amplitude versus full‐width‐at‐half‐maximum (FWHM) for three example subjects (young, age 29, blue; middle, age 59, green; old, age 64, red). (B) Normalized tuning curves for all subjects, color coded by age. (C) Tuning curves averaged across the three age groups (young, blue; middle, green; old, red). Shaded regions are SEM (standard error of mean) across sessions. (D) Tuning widths vary significantly with age, increasing for pHRFs and decreasing for nHRFs.

**FIGURE 8:**
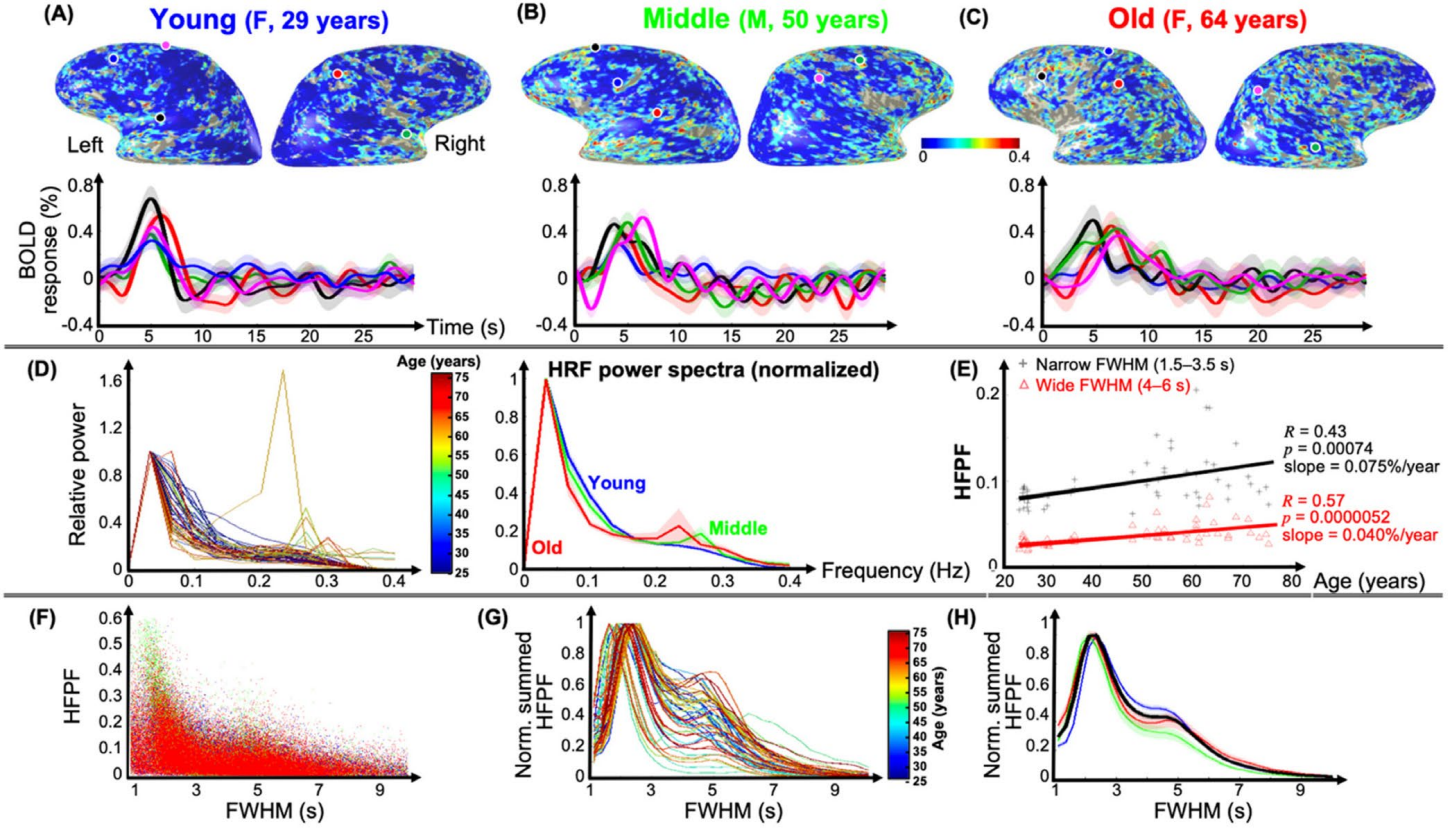
Top: HRF high‐frequency power fraction (HFPF) maps overlaid on gray‐white interface surfaces for (A) 29‐year‐old female (young), (B) 50‐year‐old male (middle), and (C) 64‐year‐old female (old). Second row: Average HRFs (5‐mm gray‐matter disk) in five example ROIs with large HFPF for each subject. Shaded regions show SEM. Third row: (D) Left plot shows normalized HRF power spectra for all 55 sessions color coded by age. Right plot shows the average of normalized HRF power spectra across sessions in three age groups: Young (blue), middle‐aged (green), and older (red). (E) Correlations between HFPF and age for narrow (1.5–3.5 s) and wide (4–6 s) FWHM for all subjects are both strong and highly significant. Bottom: Tuning curves for HFPF. (F) Plotting of HFPF versus FWHM shows a strong association between large HFPF and narrow FWHM. (G) This tuning peaks at a FWHM value of 2.3 s, with a lower peak or shoulder near a FWHM of 5 s. (H) This tuning pattern does not vary much with age. Shaded regions in all plots show SEM across sessions. FWHM: Full‐width‐at‐half‐maximum, SEM: Standard error of mean.

We first show the results of correlations between age and the spatial mean (Figure 3) and the spatial variability (Figure 4) of five HRF parameters: CNR, peak amplitude, TTP, FWHM, and HFPF for both pHRFs and nHRFs. Next, we elaborate on those HRF parameters that significantly correlated with age (Figures 5, 6, 7, 8).

Figures 3 and 4 show correlations between age and the spatial means and standard deviations, respectively, of pHRFs' (first rows) and nHRFs' (second rows) parameters. In all plots, a thick red regression line indicates a significant (*p* < 0.05) age‐related trend.

For pHRFs, mean CNR significantly (*R* = −0.41, *p* = 0.0086) decreased with age. There was also a strong, significantly (*p* < 0.05) negative correlation between CNR spatial variability and age (*R* = −0.59). Consistent with the CNR drop, peak amplitude, also showed a trend for age‐related decrease, although this was not significant (*R* = −0.22, *p* = 0.25). Peak amplitude variability also significantly dropped with age (*R* = −0.48, *p =* 0.0019).

We tested for correlations between task performance and peak amplitude and CNR but found no significant trends. Task accuracy, for example, had age correlations of *R* = −0.083 (*p* = 0.55) for amplitude and *R* = 0.006 (*p* = 0.95) for CNR. Similarly weak and insignificant correlations were observed for the validity with mean peak amplitude and CNR, and likewise for the standard deviations of the peak amplitude and CNR distributions.

Dynamical parameters show three strong age‐dependent correlations. First, the spatial variability of FWHM significantly increased with age (*R* = 0.52, *p* = 0.0006). Additionally, there were substantial increases in both the mean and spatial variability of HFPF with age (*R* = 0.44, *p* = 0.0059, and *R* = 0.38, *p* = 0.0055).

Spatial smoothing did somewhat affect these correlations (Figures S2 and S3). Smoothing weakened the downward trend of CNR with age to −0.025/year, making it insignificant (*p* = 0.16). The downward trend of mean peak amplitude also weakened. However, the significant dynamical trends in FWHM variability, and HFPF mean and variability were largely unaffected by smoothing.

For nHRFs, as shown in the second (bottom) rows of Figures 4 and 5, our results did not show any significant age trends in the moments of the CNR distributions. Dynamically, there were several significant trends. Similar to the pHRFs, mean HFPF increased significantly with age (*p* = 0.0017). Likewise, nHRF spatial variability increased for FWHM (*p* = 0.046) and HFPF (*p* = 0.0063). In addition, there was a significant trend for the spatial variability of TTP to increase with age (*R* = 0.34, 0.011/year, *p* = 0.013). Smoothing again had little effect on these trends for nHRFs (Figures S2 and S3).

We further investigated the spatial variability of HRF peak amplitudes across three groups of young, middle‐age, and old. Figure 5 shows peak amplitude histograms of strong pHRFs and nHRFs for three example subjects, each representative of an age group: a 29‐year‐old female adult for the young group (Figure 5A), a 50‐year‐old male for the middle‐age group (Figure 5B), and a 64‐year‐old female for the old group (Figure 5C). For each subject, on overlay of peak amplitudes is shown on cortical surfaces below the corresponding histogram. The overlays for the young subject show greater spatial prevalence of strongly positive peak amplitudes (orange areas), which become less prevalent for the middle‐aged subject (Figure 5B), and least for the older subject (Figure 5C). This age trend is also evident from comparison of their histograms (first row in Figure 5A–C). Thus, the reduction of pHRF peak amplitude variability with age appears to be associated with a moderation of response amplitudes in sensory regions such as lateral occipital (visual) and superior temporal (auditory) areas. This effect is also evident in normalized distributions of peak amplitude averaged across the three age groups (Figure 5D). For pHRFs, the distributions show a small shift of the mode to the right, while a “tail” of strong activation decreases. These two age‐dependent effects, which are subtly evident in Figure 5D, can be quantified by examining the behavior of quintiles of the amplitude distribution (Figure S4A). The upward trend in the mode is seen in the significant (*p* = 0.016) positive trend in the 20th percentile.

For nHRFs, the mean distributions of peak amplitudes show a simpler behavior, with the distribution steadily shifting toward greater (more negative) amplitudes with increasing age. Quantifying by quintiles (Figure S3B), there are significant downward trends in 40th, 60th, and 80th percentiles.

As mentioned above, regression showed a substantial increase in FWHM spatial variability with age (Figure 4), with a stronger correlation for pHRFs (*R* = 0.52) than nHRFs (*R* = 0.27) (Figure 4). These results are further understood by examining the FWHM distributions (Figure 6). FWHM distributions of strong HRFs and their corresponding maps overlaid on gray‐white interface surfaces are shown for the same representative subjects used for Figure 5. Average HRFs (5‐mm‐diam gray‐matter disk) in six example ROIs are plotted for each subject. The young subject (Figure 6A) had a relatively narrow, unimodal pHRF FWHM distribution with a peak at 4.7 s. The overlay of FWHM on the cortical surface is consistent with the fairly stable dynamics reported previously (Taylor, Kim, and Ress 2018). Example HRFs illustrate stereotypical HRF behavior. However, the FWHM distribution for the middle‐aged adult was notably broader (Figure 6B), with many narrower HRFs across cortex evident on the overlay. Example HRFs illustrate the mixture of stereotypically broad as well as narrower HRFs. The FWHM distribution became even broader for older adults, showing a greater number of narrower HRFs across the cortex (Figure 6C). The distribution was strongly bimodal, with a clear diversity of FWHM values evident on the surface overlay. Example HRFs illustrate these narrow and broad classes of HRFs. FWHM distributions from all subjects were normalized by their peak values and averaged together for young (< 40 years), middle (40–59 years), and old (≥ 60 years) groups (Figure 6D). Clearly, pHRFs with lower FWHM values are less likely in the young group (blue distribution), becoming more common with advancing age, so that the older group shows a bimodal distribution (red distribution), with a distinct mode of narrow HRFs, as well as a greater representation of unusually broad HRFs. Examination of FWHM distributions for all subjects (Figure S5) confirm this tendency toward broader, bimodal distributions with increasing age.

Scatter plots of peak amplitude versus FWHM show that largest amplitudes are associated with a fairly narrow temporal range for both pHRFs and nHRFs (Figure 7A) in the same three example subjects used previously. Tuning curves obtained from this data emphasize this feature and show strong changes with age (Figure 7B). When these curves are averaged over our three age groups, the age‐related changes become clear (Figure 7C). For pHRFs, tuning is sharpest for the young group (blue), becoming broader for the middle (green) and older (red) groups. Moreover, the old group exhibits a tendency toward a second peak of faster dynamics. For nHRFs, the behavior is reversed, with tuning broadest for the young group and becoming sharper for both middle and older groups with a possible second mode of slower dynamics emerging for the older group. The changes in tuning width indeed vary significantly with age (Figure 7D) for both pHRFs (*R* = 0.49, *p* < 10^−4^) and somewhat more weakly for nHRFs (*R* = 0.29, *p* = 0.032).

Next, we found a significant association between the age and the HFPF of both pHRFs and nHRFs for both spatial mean (Figure 3) and standard deviation (Figure 4). In Figure 8, these age‐related trends are illustrated for the same representative subjects used previously. Cortical areas with strong HFPF were lowest for the young subject (note prevalence of dark blue clusters in Figure 8A), becoming greater for the middle‐aged subject (Figure 8B, increasing prevalence of cyan clusters) and greatest for the older subject (Figure 8C). Sample HRFs show the character of the high‐frequency oscillations in these three subjects.

HRF power spectra (without upsampling) were normalized by their peak value at frequencies < 0.1 Hz for all 55 sessions (Figure 8D). All but one session show spectra that peak at a low frequency with a tail toward higher frequencies. However, for many middle‐aged and older subjects, the spectrum also features a smaller peak at a relatively high frequency (> 0.2 Hz). Notably, one session (female, age 58) showed an atypical spectrum with a larger peak at high frequency. When these normalized spectra were averaged over our three age groups (Figure 8D, middle), the low‐frequency spectrum was narrowest for older (red), then middle‐aged (green), and broadest for the young (blue). In addition, the power spectra for both middle‐age and old groups show a distinct second peak at high frequency.

The trend toward narrower FWHM and greater HFPF are necessarily linked. To investigate, we tested whether the trend between the HFPF and age was different for pHRFs with FWHM in narrow‐mode range (1.5–3.5 s) versus those in the wide‐mode range (4–6 s) across all subjects (Figure 8E). For both, correlations between HFPF and age were highly significant. Notably, the age trend was stronger for the narrow‐mode FHWM HRFs (slope = 0.075%/year vs. 0.04%/year), but the correlation was stronger for the wide‐mode FWHM HRFs (*R* = 0.57, *p* = 0.000005 vs. *R* = 0.43, *p* = 0.0007). Moreover, scatter plots of HFPF versus FWHM suggest that large HFPF is associated with narrow FWHM (Figure 8F). Associated tuning curves confirm a dominant peak near 2.3‐s FWHM, with a lesser peak or shoulder near 5‐s FWHM (Figure 8G). This qualitative pattern shows little age dependence, but there is a significant decrease with age in HFPF near the 5‐s shoulder (Figure 8H).

Motivated by the results of West et al. (2019), we investigated if onset time mean and variability would change globally with age (Figure S6). Here, we define onset time as the time to the half maximum. We did not find any significant age trend in the spatial mean of onset time for both pHRFs and nHRFs, or in the spatial variability for pHRFs. However, for nHRFs, onset‐time spatial variability significantly increased with age.

Motivated by our previous work (Taylor, Kim, and Ress 2018), we evaluated age‐related changes in four correlations between pHRF parameters: TTP and FWHM, TTP and time‐to‐undershoot (TTU), peak amplitude and undershoot, TTU and FWHM (Figure S7). For the current dataset of 55 sessions, we confirmed that these correlations were again present and significant (*p* < 0.0001). TTP versus FWHM correlations did not show any significant age‐related dependence. TTP versus TTU correlations, modestly but significantly weakened with age (*R* = −0.317, *p* = 0.0155). Similarly, peak amplitude versus undershoot correlations also showed a pronounced weakening with age (*R* = 0.526, *p* = 0.0007). Finally, TTU versus FWHM correlations, moderately but significantly increased with age (*R* = 0.353, *p* = 0.0222).

Additionally, for each session, we performed principal component analysis (PCA) on strong (CNR > 3) pHRFs and nHRFs, separately. The first PCA component, the mean HRF, was then parameterized for peak amplitude, TTP, FWHM, and HFPF. We then performed a regression for each parameter against age. The results revealed a significant increasing trend for two pHRF parameters (Figure S8): FWHM (*R* = 0.34, *p* = 0.039) and HFPF (*R* = 0.27, *p* = 0.045).

Finally, we further investigated if correlations between HFPF and peak amplitude would be associated with age for (1) both pHRFs and nHRFs in full range of FWHM, (2) pHRFs with FWHM in narrow‐mode range (1.5–3.5 s) versus those in the wide‐mode range (4–6 s), and (3) nHRFs with FWHM in narrow‐mode range (1.5–3.5 s) versus those in wide‐mode range (4–6 s) (Figure S9). First, note that the correlations are generally negative for pHRFs, and positive for nHRFs. Thus, stronger HRFs generally exhibit less HFPF. However, these correlations are significantly stronger (*p* = 0.0001) for the wide‐FWHM HRFs than for the narrow‐FWHM HRFs. Also, these correlations weaken with age (Figure S9A). For pHRFs, the negative correlations had a significantly increasing trend with age only for those with FWHM in narrow‐mode range (*R* = 0.30, *p* = 0.025). However, only nHRFs with FWHM in wide‐mode range decreased significantly with age (*R* = −0.29, *p* = 0.04; Figure S9B).

## 4 Discussion

We provided a detailed evaluation of age‐related HRF trends using an experimental protocol that activates the majority of cerebral cortex. High‐resolution fMRI acquired this data with minimal partial‐volume effects in the native space of a group of subjects with ages between 22 and 75 years. We found significant changes in the distributions of both HRF amplitudes and, more importantly, dynamics. Our main findings are: (1) there is a significant trend for mean CNR to decrease with age, but this trend is substantially reduced when data are smoothed to 8‐mm FWHM. (2) The spatial variability of peak amplitude and CNR significantly decrease with age. (3) Dynamically, the strongest age‐related trend is an increase in FWHM spatial variability with age. (4) HRF amplitudes tend to be narrowly tuned to a particular range of FWHM values, and the tuning width increases significantly with age. (5) There is a significant increase in HFPF mean and standard deviation with age, and the HFPF is tuned to narrow FWHM values. The results document how age systematically changes the dynamics of NVC in a fashion consistent with known changes in the cerebral microvasculature.

In the present work, the SAST (Taylor, Kim, and Ress 2018, 2022) enabled us to globally examine age‐related changes in the HRF for all subjects. Our study is, therefore, notably different from previous studies that were only focused on specific brain regions (e.g., motor and vision areas) (Aizenstein et al. 2004; West et al. 2019; Ross et al. 1997; Ances et al. 2009; Buckner et al. 2000; Raemaekers et al. 2006; Morsheddost, Asemani, and Alizadeh Shalchy 2015). The suitability of the SAST for a whole‐cortex study of hemodynamic responses was indeed confirmed by our results demonstrating strong (CNR > 3) HRFs in 60%–90% of cerebral cortex (average 68% ± 8% at full resolution, 87% ± 8% after smoothing to 8‐mm FWHM). For those strongly activated areas, the majority of responses were pHRFs (average 73% ± 8%, Figure 2D). Additionally, the fixed interstimulus intervals used in the SAST allowed us to intensively average over ~80 time‐locked events. This averaging approach provided an excellent rejection of random‐phase nuisance sources such as pulsatility and respiration (Taylor, Kim, and Ress 2018, 2022) despite poor estimation efficiency compared to tasks with a randomized design (Liu et al. 2001).

The distributions of pHRF amplitudes were significantly affected by age in two ways. First, evaluation of the distributions by quintiles (Figure S4) showed two distinct effects for pHRFs: weaker amplitudes significantly increase with age, while strong amplitudes significantly decrease with age. Qualitatively, the statistical mode of the pHRF amplitude distributions increase with age, while the tail of strong amplitudes diminishes with age. Second, spatial variability of pHRFs strongly and significantly decreased with age (Figure 4). The pHRF results suggest that NVC becomes more conservative with aging, with a paucity of high‐amplitude HRFs, consistent with previous reports (Ross et al. 1997; Ances et al. 2009; Buckner et al. 2000; Raemaekers et al. 2006; Morsheddost, Asemani, and Alizadeh Shalchy 2015). For nHRFs, the behavior is simpler, with all HRFs becoming stronger (more negative) with age, but this effect is clearest for the weakest nHRFs. Taken together with the decrease in nHRF fraction, this suggests more conservative allocation of NVC suppression with aging.

Amplitudes and CNR may have been affected by task performance and head motion. Task performance significantly dropped with age (Figure 2A,B). Accuracy and validity were probably lower for older adults because they tended to respond too slowly, so that their responses referred to the previous firework display. Indeed, for correct trials, we found larger reaction times for the older adults (Figure S1). However, when participants made an error, the reaction time was typically very short, indicating that these responses corresponded to the previous firework display. The decreased performance may have impacted pHRF CNR, which showed a significant downward trend with age (Figure 3). However, no correlations were found with measured task performance metrics. This decrease in CNR may also have been caused by greater head motion in the older subjects, so that motion censoring decreased the number of events available for analysis. However, when we removed this effect by scaling the CNR values up by the square‐root of available events, the correlation of CNR with age was not significantly altered. Thus, neither performance nor head motion seems to have played a dominant role in the age‐related decreases in peak amplitude and CNR. Interestingly, these downward trends weakened with 8‐mm FWHM smoothing (Figure S4), suggesting that the CNR degradation is spatially fine grained. This, however, stands in contrast to the other parameter variations with age, which were unaffected by smoothing (Figures S4 and S5). Apparently, smoothing reduces the CNR loss associated with aging, but does not much affect the age trends of the HRF dynamical parameters.

We also examined the effect of aging on HRF dynamics. Our results showed that the spatial variability of FWHM strongly and significantly increases with age (Figure 4). Moreover, comparison of distributions averaged over young, middle‐aged, and older groups (Figure 6D) show a broadening of the overall distribution as well as the emergence of a distinct mode of narrow‐FWHM HRFs. However, a detailed examination of FWHM distributions for all subjects (Figure S5) indicated that two distinct modes were not always found in older subjects, while a bimodal distribution was also present in a few middle‐aged adults. This pattern of results suggests that the associated changes in NVC do not occur in all older subjects. Further experiments are needed to assess the relationship of these dynamical changes to pathologies such as hypertension.

Such findings, to our knowledge, have not previously been reported. In a study by West et al. (2019), the averaged HRFs for each occipital, temporal, left hemisphere precentral lobes did not show any significant differences in FWHM between younger and older groups (West et al. 2019). In another study by Zysset et al. (2007), an increase in HRF FWHM was only found in the inferior frontal junction and the inferior fusiform gyrus for older versus younger groups (Zysset et al. 2007). It is possible that such changes are not broadly evident when data are evaluated in a standard space or after averaging across large ROIs because the spatial patterns of these narrow FWHM occurrences are not common across subjects. The examples in Figure 6 support this idea, but this hypothesis needs more extensive testing.

Our previously developed model can conceptually connect these changes in pHRF dynamics with known age‐dependent microvascular disruptions within the context of linear‐systems analysis. Specifically, in the healthy young brain, the pial arterioles provide a pressurized, highly interconnected mesh that enables NVC, thereby creating the HRF. Disruption of this mesh by arterial rarefaction or lipohyalinosis could lead to both faster and slower dynamics. Faster dynamics may correspond to a loss of linkage between pial‐mesh elements, reducing the available compliance and arterial blood volume needed to drive NVC. Compliance might also be directly reduced in arteriolar segments due to wall thickening and a decrease in luminal diameter. In other words, mesh disruption reduces the stored hemodynamic energy available to respond to a proximal integration event. Such faster and weaker dynamics would correspond to GM regions with a viable penetrating arteriole connection to a disrupted portion of the pial mesh. Conversely, slower dynamics could correspond to GM regions without a direct connection to the pial mesh. These regions might still manifest NVC by blood flow among superficial arteriole anastomoses (Baran, Li, and Wang 2015; Duvernoy, Delon, and Vannson 1981; Luo et al. 2017). The longer path lengths for such NVC could contribute to the observed broadening of the HRF FWHM. Such collateral circulation mechanisms have been associated with compensation for focal ischemia from stroke, but they could also provide NVC in the context of age‐related disruptions to the pial mesh.

Changes to neurometabolic coupling could also explain the narrower FWHM HRFs that emerge with aging. Specifically, stronger early CMRO_2_ could reduce the FWHM. However, predictions from our previously developed modeling work (Kim and Ress 2016) suggest that such changes in metabolism would have to be quite large (> 40%). Moreover, such a change should also produce a global increase in onset time, which was not observed (Figure S6). We therefore tentatively conclude that neurometabolic changes are unlikely to be the cause of the narrower FWHM for pHRFs, but further measurements and modeling are needed to explore this issue.

Our data showed a marginal trend (*p* = 0.081) of decreasing negative fraction of cortical coverage with age (Figure 2C). Because the majority of the measured nHRFs likely correspond to deactivation of the default mode network (DMN), this may be related to previously noted age‐related reductions in DMN activity (Mevel et al. 2011; Hafkemeijer, van der Grond, and Rombouts 2012). However, in contrast to pHRFs, nHRFs were observed to become more tightly tuned with age (Figure 7). It may be that for healthy subjects the narrower tuning of nHRFs with age corresponds to more efficient NVC that compensates for a smaller cortical area of deactivation in order to sufficiently provide DMN functionality (Hafkemeijer, van der Grond, and Rombouts 2012).

We also report, for the first time, high‐frequency oscillations in the HRF that increase with age. Such findings were not anticipated and therefore, represent new observations not originally hypothesized. One possible explanation is an age‐related increase in respiratory noise. If so, our results provide a cautionary note regarding the interpretation of fMRI in older subjects. However, the observed dynamics are inconsistent with respiratory noise for several reasons. Our analyses were limited to voxels with strong (CNR > 3) HRFs. Moreover, the power spectra for many older and middle‐aged adults demonstrated a distinct peak in range of 0.2–0.4 Hz, consistent with a coherent oscillatory feature. The consistent oscillations that are evident after averaging over ~80 events could not be manifest by random‐phase noise or physiological nuisance (Taylor, Kim, and Ress 2022). Indeed, the time‐locked averaging approach has the ability to filter out the highly structured noise. In particular, power spectra for respiratory noise and physiological nuisance tend to be a broad‐band phenomena rather than narrow, distinct peaks as shown in Figure 8D (Glover et al. 2000). Furthermore, our HRFs showed globally high CNR, again suggesting that time‐locked averaging approach provides very good rejection of high‐frequency nuisance like pulsatility and respiration (Taylor, Kim, and Ress 2022). Finally, there is no immediate physiological interpretation as to why respiratory noise should increase with age. Such noise is driven by respiratory susceptibility changes in the chest as the lungs inflate and deflate. Most age‐related pulmonary pathology decrease lung volume and would be expected to reduce respiratory noise. Thus, the advent of greater HFPF with age appears to indicate a new age‐related dynamic. These results may provide a hemodynamic basis for the findings of previous studies exploring age‐related changes in the frequency content of resting‐state fMRI signals (Zhong and Chen 2022; Yang et al. 2018). In particular, Zhong and Chen (2022) demonstrated spatially extensive increases in the 0.1–0.3‐Hz range.

We conducted a supplementary analysis to further investigate the spectral character of the noise (and nuisance) in our data in a subset of 8 of our subjects where segmentations of area V1 were available (Figure S10). For each vertex, we took the depth‐averaged time series (zero‐mean data) and subtracted a 16‐fold replicated set of session‐mean HRFs, also with temporal mean removed. Note that this procedure should reveal not only nuisance effects such as respiratory and cardiac pulsation, but also trial‐to‐trial variability in the HRF itself. We then calculated the power spectrum at each vertex with CNR > 3 and averaged across vertices. Because the V1 data were predominantly pHRFs, we ignored the nHRFs. The calculation was performed for each run. The mean spectrum across runs, together with its SEM, is shown for all subjects in the top eight plots. All spectra show the same basic character, with interesting structure at low frequencies (< 0.13 Hz), but becoming mostly flat and featureless at higher frequencies. When these spectra are averaged across subjects, the pattern is very similar. Because respiratory nuisance typically occurs in the range of 0.2–0.4 Hz, the featureless spectra we observe confirms its effective removal by our time‐locked averaging procedure. Nuisance from cardiac pulsatility is more difficult to evaluate, because it was not resolved by our 1.25‐s TR. Nevertheless, the random‐phase averaging should be even more effective for this higher‐frequency (~1 Hz) noise. Finally, the low‐frequency spectra then likely correspond to hemodynamic variability in the NVC that gives rise to the HRF, and this could provide another interesting observable for further age‐related analysis in future work.

These spectral HRF features could reflect fundamental modes of the pial mesh. From a linear‐network viewpoint, when the mesh is intact and roughly balanced, many of the poles and zeroes of the network cancel out, leaving the observed low‐frequency dynamics that are tuned to a FWHM near 5 s. When the mesh is abrogated by aging or age‐related pathology, faster oscillations could emerge. The tuning of the oscillations toward the narrow‐FWHM dynamics with age may be consistent with a weakly damped flow response suggested by our previous modeling (Kim and Ress 2016; Kim et al. 2013). Further experiments and simulations will be needed to test this hypothesis.

As shown in Figure S7, our evaluation of HRF parameter correlations with age revealed an age‐related decrease in correlations between peak amplitude and undershoot. Such a finding is consistent with general weakening of the hemodynamic response and the HRF peak amplitudes in older subjects. Peak amplitudes and undershoot highly correlate with each other but the weakening of these amplitudes with age would therefore result in a decrease in their correlation. Similarly, the weakening of TTP versus TTU correlations with age may also be a result of increased temporal uncertainty as amplitudes decrease. However, despite amplitude effects, correlations between TTU and FWHM increased with age. In earlier work, we hypothesized that both peak and undershoot are primarily driven by CBF (Kim and Ress 2016; Breuer et al. 2005), but they are also modified by CMRO2. Accordingly, an increase in the correlation between TTU and FWHM could imply less variability in metabolism with age, consistent with the observed reduction in both spatial mean and variability of peak amplitude with age. This hypothesis remains to be further explored in future experiments.

We generally observed an inverse correlation between HFPF and peak amplitude (Figure S9). This suggests that stronger HRFs, which are most likely demonstrative of healthy NVC, are less associated with HFPF, which could be a manifestation of NVC pathology. Moreover, the two FWHM modes of the HRF, narrow and wide, that become more prevalent in older subjects, show different correlations between HFPF and peak amplitude. In particular, pHRFs with narrow FWHM show generally weaker correlations between amplitude and HFPF, and these further significantly weaken with age (Figure S9. A), consistent with the idea that narrow‐FWHM HRFs are also a manifestation of NVC pathology.

We also report the results of regression analyses on four parameters from the first PCA component, the spatial mean HRF (Figure S8). The age‐related findings from this analysis were largely consistent with those from the spatial mean of HRF parameters, showing no significant trends for peak amplitudes or TTP, and upward trends for HFPF, although the latter were only significant for pHRFs. However, there was a significant increase in FWHM with age for pHRFs. Such a result is consistent with the age‐related broadening of mean normalized FWHM distributions. Therefore, the PCA mostly confirmed the results of our spatially global analysis.

Our study has several limitations. First, we did not deal with sex differences among our subjects. In fact, our subject pool was not well sex balanced, with more females than males. Future work will be necessary to evaluate this issue. Second, our analyses were deliberately global in the native space of individual subjects. Further analysis to evaluate spatially dependent effects in a standard space would be useful. Third, the SAST evokes HRFs across only a majority of cortex. This “window” into brain function, varies somewhat across subjects (Taylor, Kim, and Ress 2018) adding a small confound to the age‐variability data. Also, for some older subjects, a slower version of the SAST was used, causing a possible confound to the data. However, all subjects of our study performed the task for the same duration and thus, we expect similar evocation the HRFs in all subjects. Fourth, the demanding audio‐visual task during each event is followed by a less‐demanding color‐detection fixation task, so the change in vigilance state may confound the HRFs (Li et al. 2016). Fifth, we used a large dataset to evaluate a total of 20 age‐dependent effects (2 moments [mean/standard‐deviation] × 5 parameters × 2 HRF types [negative/positive]). Accordingly, there is a possibility for false positives, but an appropriate correction is non‐obvious. Therefore, caution should be taken in the interpretation of our exploratory outcomes and these results should be viewed as hypothesis generating and needed to be confirmed by future work (Kim et al. 2020). Sixth, our time‐locked averaging approach was conducted on the events that survived head motion (Table S1); therefore, there was uneven weighting across subjects, with a smaller amount of data for some individuals, particularly older adults. Finally, these data relied on BOLD contrast to evaluate age effects. Further experiments with time‐resolved HRF flow measurements (Rangaprakash et al. 2021) would be desirable.

Finally, several research groups have suggested fMRI approaches to evaluate various neurodegenerative pathology (Rangaprakash et al. 2017; Yan, Rangaprakash, and Deshpande 2018; Asemani, Morsheddost, and Shalchy 2017; Krishnamurthy et al. 2021; Song et al. 2023; Mitchell et al. 2011). Our study also provided evidence suggesting that various HRF parameters, particularly FWHM and HFPF, have promise as indicators of age‐related pathology. In particular, normal aging has been associated with increases in flow pulsatility due to mismatch between peripheral arterial stiffness and aorta stiffness (Xu et al. 2017a; Xu et al. 2017b; Tarumi and Zhang 2018). Such changes are exacerbated by hypertension, which occurs increasingly with age. The pulsatility can damage the cerebral microvasculature. Therefore, we hypothesize that excessive flow pulsatility in older adults contributes to disruption of the pial mesh, consistent with weaker, more oscillatory HRFs with both narrower and broader FWHM. Future work is needed to assess whether these dramatic age‐related changes in HRF dynamics and NVC are associated with hypertension or other age‐related pathologies. Nevertheless, use of HRF evoked by the SAST already shows great promise as a non‐invasive metric for age‐related pathology.

## Author Contributions

N.J.F.: conceptualization, data curation, formal analysis, investigation, methodology, validation, writing (original draft and preparation), writing (review and editing). A.T.: conceptualization, data curation, formal analysis, methodology, validation, writing (review and editing). K.M.: data curation. j.h.k.: data curation, methodology, resources, supervision, writing (review and editing). D.R.: conceptualization, data curation, formal analysis, investigation, methodology, validation, project administration, resources, supervision, validation, writing (review and editing).

## Ethics Statement

The authors assert that all procedures to this work comply with the ethical standards of the relevant national and institutional committees on human experimentation and with the Helsinki Declaration of 1975, as revised in 2008.

## Conflicts of Interest

The authors declare no conflicts of interest.

## Supporting information


Data S1.

## Data Availability

The derived data that support the findings of this study are available on request from the corresponding author, David Ress. The experimental MRI data deidentified as required by our IRB protocol, can also be made available in the form of the DICOM image sets upon request. All software used in our analysis is part of mrHirbil package, which is also available for download upon request. Processed data, HRF time series and their parameters, mapped to native‐space vertices in NIFTI format, will be made available upon request to the corresponding author.
