# Supplementary material for: Global Impact of Aging on the Hemodynamic Response Function in the Gray Matter of Human Cerebral Cortex
Source: Hum Brain Mapp. 2024 Dec 18;45(18):e70100. doi: 10.1002/hbm.70100 (PMC11653092; doi:10.1002/hbm.70100)
Supplement: Supplementary file 1 — Data S1. [file HBM-45-e70100-s001.docx]

**Global impact of aging on the
hemodynamic response function**

**in the gray matter of human cerebral cortex**

**Nooshin J. Fesharaki^1,2^, Amanda Taylor^2^, Keisjon Mosby^2^, Ruosha Li^1^, Jung Hwan Kim^1^, and David Ress^2*^**

^1^Department of Neurosurgery, University of Texas Health Science Center, Houston, Texas, United States of America

^2^High Resolution Brain Imaging Lab, Department of Neuroscience, Baylor College of Medicine, Houston, Texas, United States of America

**Supplementary figures**


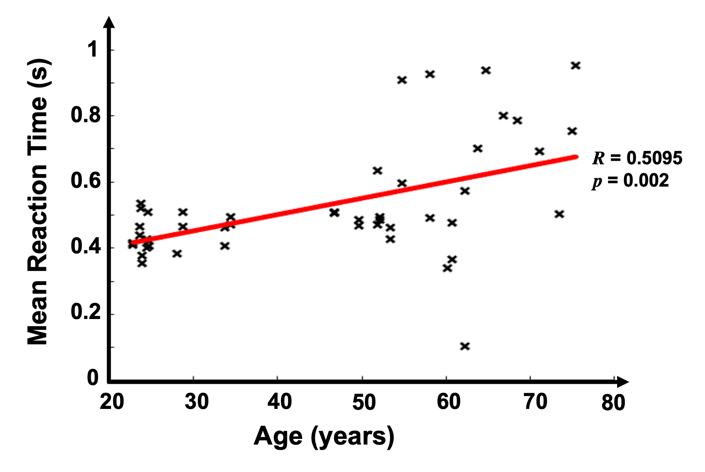


**Figure S1.** Significant (*p* < 0.05) Pearson correlation coefficient (*R*) between age and the mean of reaction times (across five scans per session).


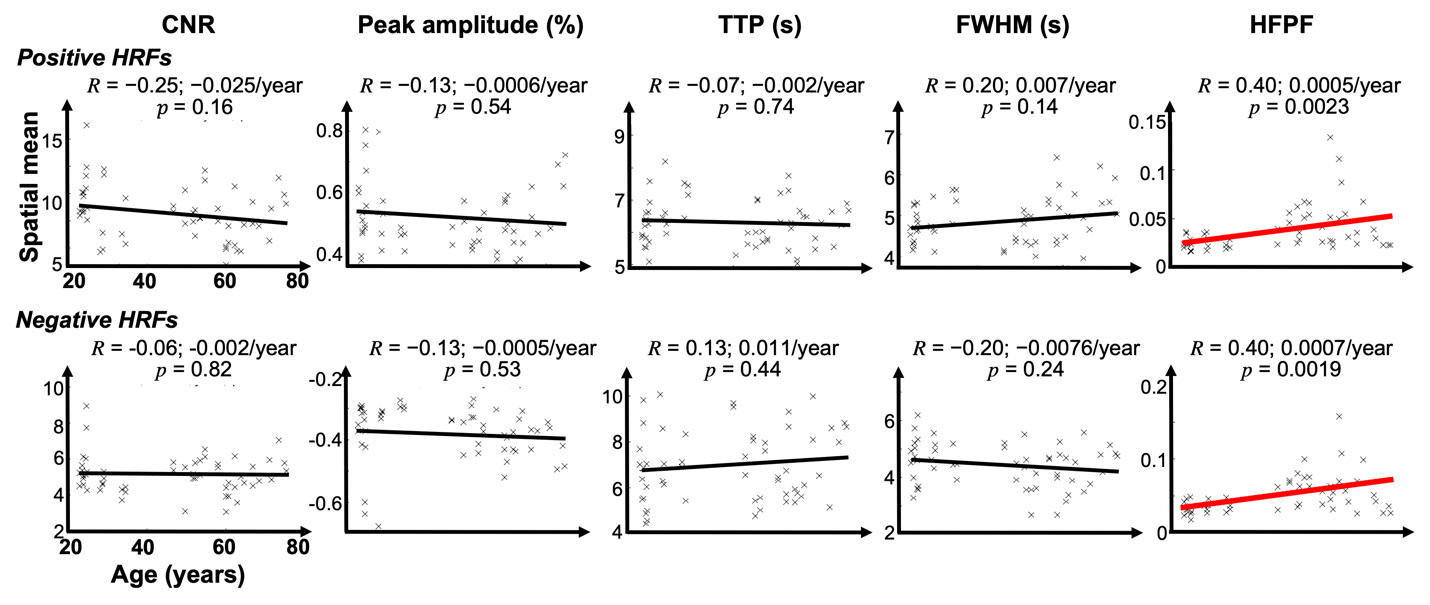


**Figure S2.** Correlations between age and the spatial mean of contrast-to-noise ration (CNR), peak amplitude, time-to-peak (TTP), FWHM, and high-frequency (HF) power fraction for top, positive HRFs (pHRFs), and bottom, negative HRFs (nHRFs) with 8-mm full-width-at-half-maximum smoothing. Pearson correlation coefficients (*R*) are shown for each HRF parameter. Significant (*p* < 0.05) correlations are marked by thicker, red regression lines.


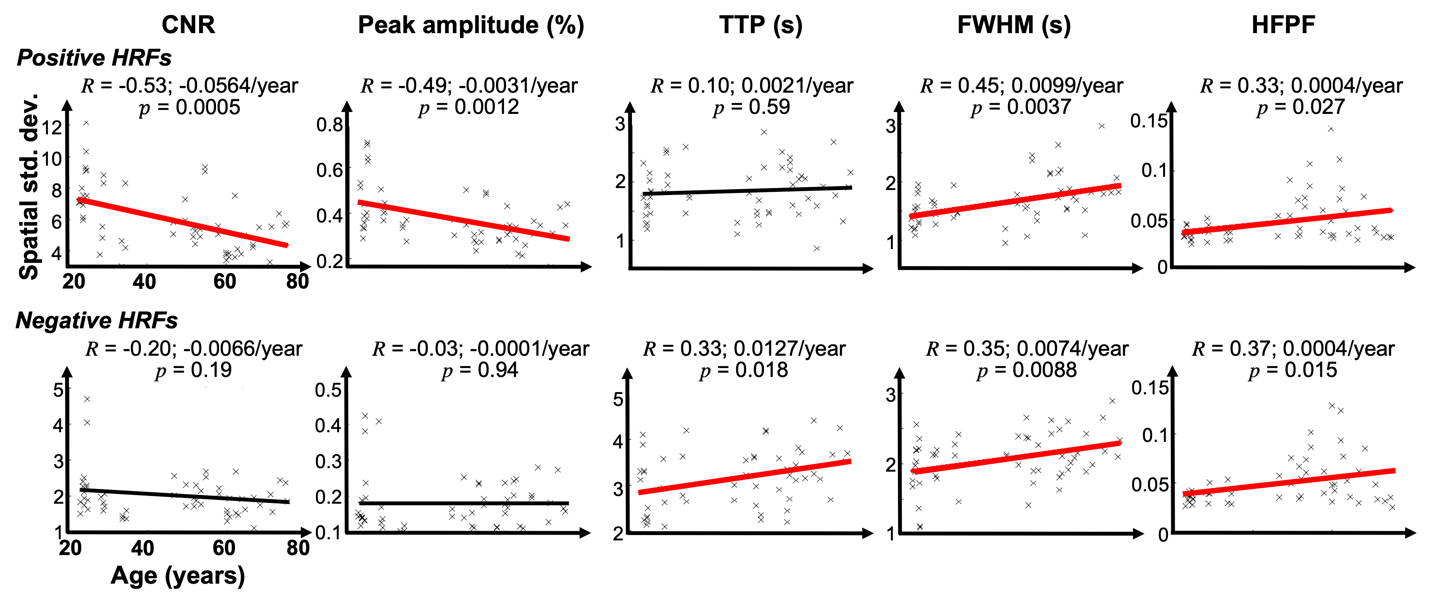


**Figure S3.** Correlations between age and the spatial standard deviation of contrast-to-noise ratio (CNR), peak amplitude, time-to-peak (TTP), FWHM, and high-frequency power fraction (HFPF) for top, positive HRFs (pHRFs), and bottom, negative HRFs (nHRFs) with 8-mm full-width-at-half-maximum smoothing. Pearson correlation coefficients (*R*) are shown for each HRF parameter. Significant (*p* < 0.05) correlations are shown by thicker, red regression lines.


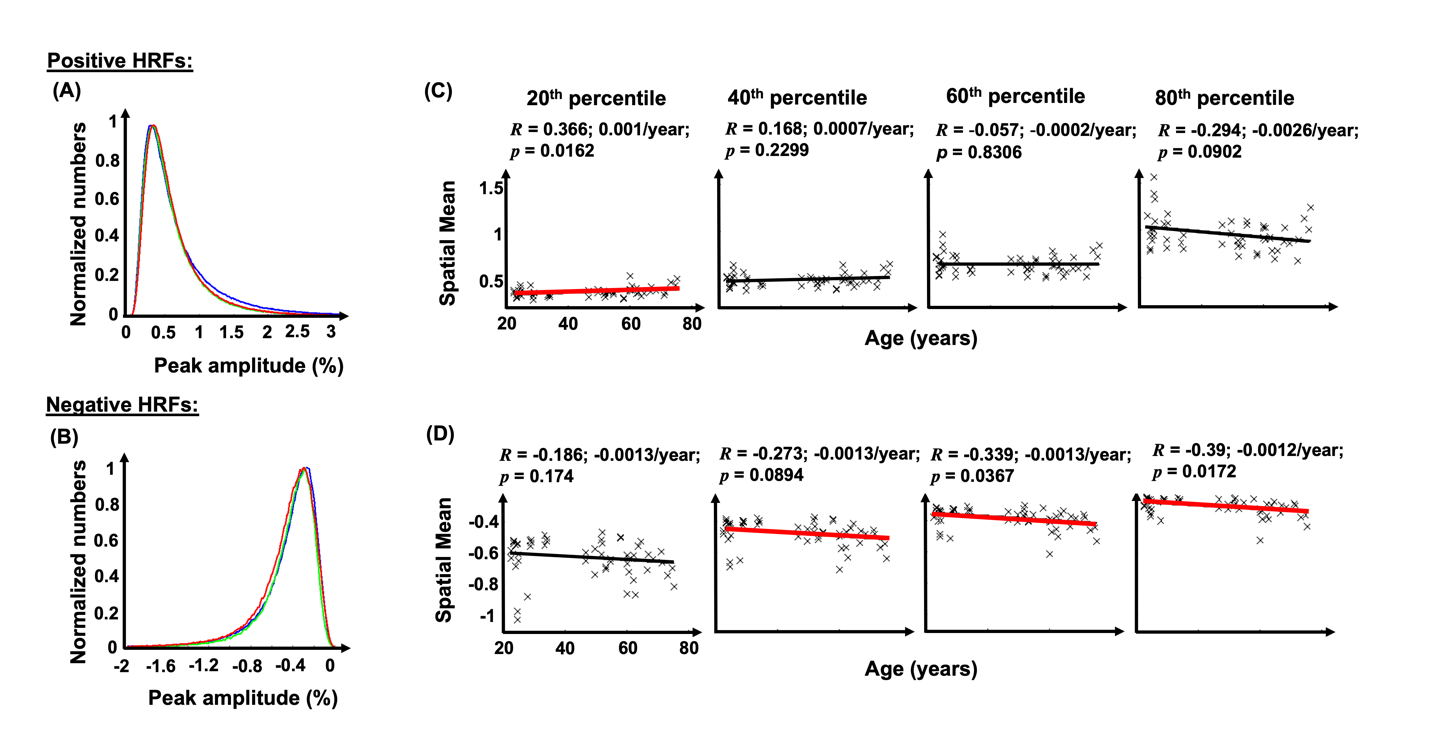


**Figure S4.** Mean normalized distributions of peak amplitude for strong (**A**) positive and (**B**) negative HRFs are shown for young (blue), middle-aged (green) and older (red) groups. Correlations between the age and the spatial mean of peak amplitudes for the 20^th^, 40^th^, 60^th^, and 80^th^ percentiles of strong (**C**) positive and (**D**) negative HRFs. Pearson correlation coefficients (*R*) are shown for association between each percentile and age. Significant (*p* < 0.05) correlations are shown by thicker regression lines in red.


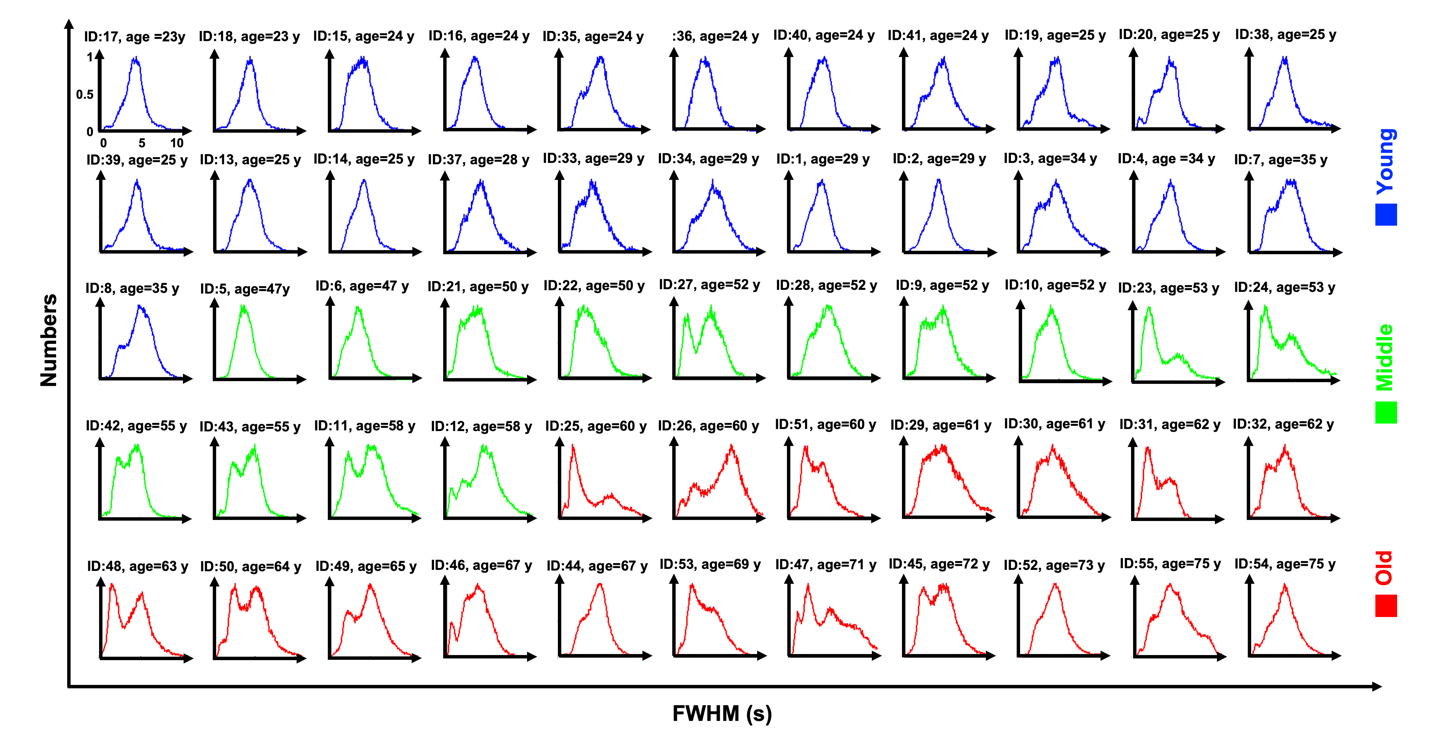


**Figure S5.** Distributions of full-width-at-half-maximum (FWHM) for strong (contrast-to-noise ratio (CNR) > 3) positive HRFs are shown for young, middle-aged, and older groups in blue, green, and red, respectively. FWHM values are in the range of 0–11 s.

**Figure S6.** Correlations between age and the spatial standard deviation of onset time for left, positive HRFs (pHRFs), and right, negative HRFs (nHRFs). Pearson correlation coefficients (*R*) are shown for each HRF parameter. Significant (*p* < 0.05) correlations are shown by thicker, red regression lines.


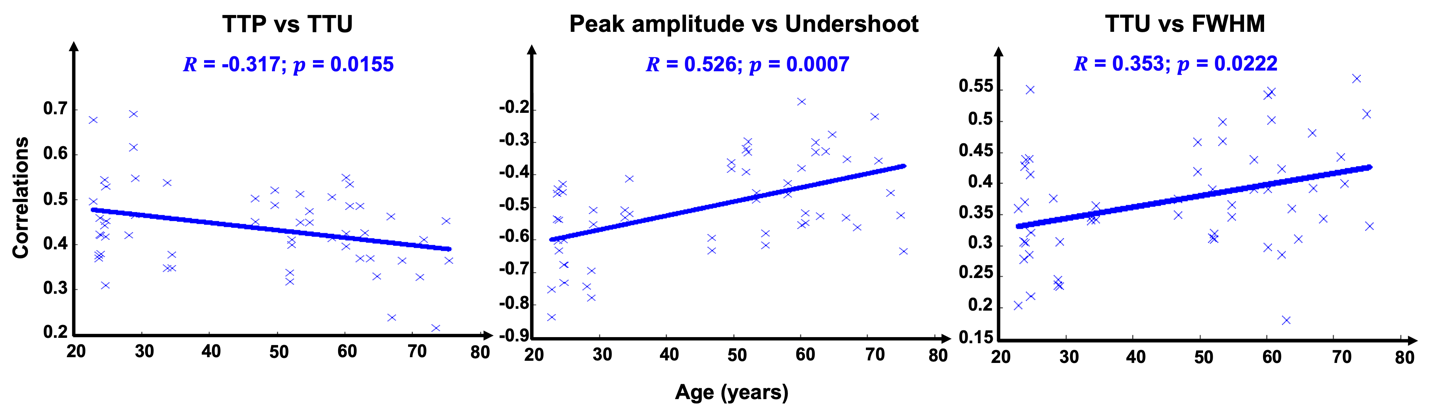


**Figure S7.** Correlations between (**left**) time-to-peak (TTP) and time-to-undershoot (TTU), (**middle**) peak amplitude and undershoot, and (**right**) TTU and FWHM with age for positive HRF. Pearson correlation coefficients (*R*) are shown for each correlation. All individual correlations between the parameters were significant at *p* < 0.0001. All three age correlations were also significant (*p* < 0.016).


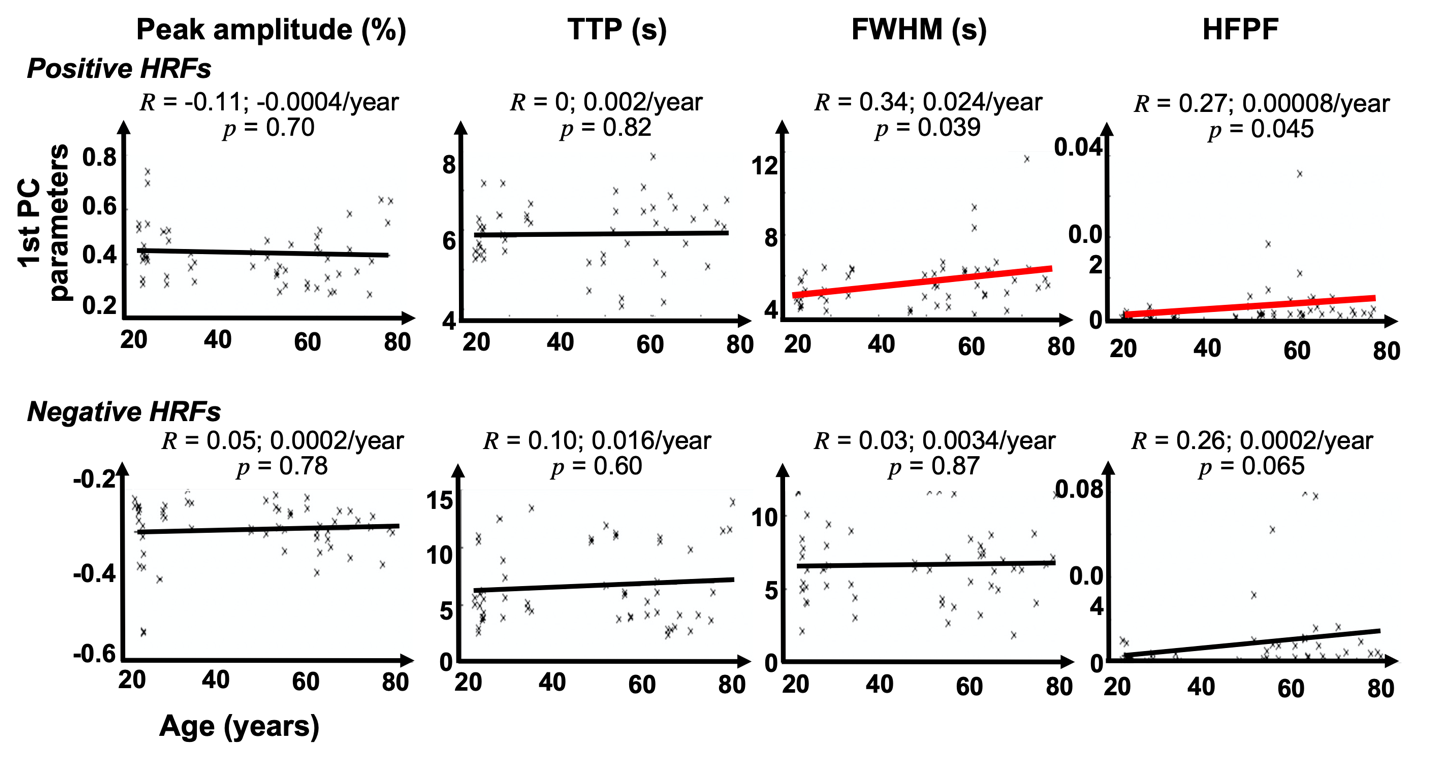


**Figure S8.** Correlations between age and the first principal component (PC) parameters: peak amplitude, time-to-peak (TTP), full-width at half-max (FWHM), and high-frequency power fraction (HFPF) for top, positive HRFs, and bottom, negative HRFs, from each session. Pearson correlation coefficients (*R*) are shown for each parameter. Significant (*p* < 0.05) correlations are shown by thicker, red regression lines.


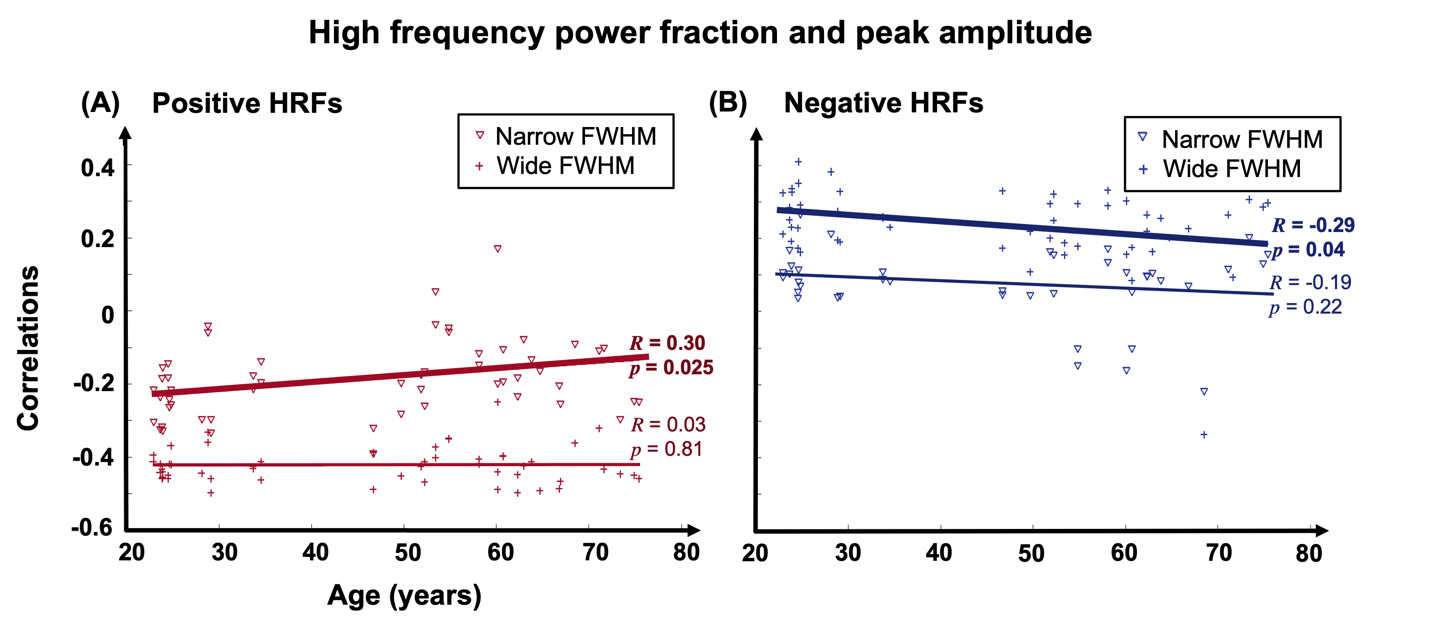


**Figure S9.** Correlations between high-frequency power fraction (HFPF) and peak amplitude as a function of age for (A) both positive HRFs and negative HRFs in full range of full-width at half-max (FWHM), (B) positive HRFs with FWHM in narrow-mode range (1.5–3.5 s) versus those in the wide-mode range (4–6 s), and (3) negative HRFs with FWHM in narrow-mode range (1.5–3.5 s) versus those in wide-mode range (4–6 s). Pearson correlation coefficients (*R*) are shown for each comparison. Significant (*p* < 0.05) correlations are shown by thicker, red or blue regression lines.

**Figure S10**. Positive HRF noise power spectra in area V1 for 8 example subjects are shown in the upper 8 plots; gray regions show SEM across runs. The noise spectra show low-frequency (<0.13 Hz) structure, and a comparatively featureless high-frequency spectrum. Bottom plot shows average across subjects, with gray regions indicating SEM across subjects. The same pattern is evident. The low-frequency noise power likely reflects variability in the HRF, while the otherwise featureless spectra confirm effective removal of physiological noise produced by respiration and cardiac pulse.

**Supplementary Table**

**Table S1.** The number of HRF events survived after censoring for head motion correction of all 55 sessions. The number of events is shown for young, middle-aged, and older groups in blue, green, and red, respectively. The total number of events is 80.
